# Long-term control of *Legionella pneumophila* and *Pseudomonas aeruginosa* in dental unit waterlines following optimization of a hypochlorous acid-based water safety program

**DOI:** 10.3389/froh.2026.1903381

**Published:** 2026-08-11

**Authors:** Alexandra Wolf, Tanya Ezat Abdullah Abdullah, Andreas Moritz, Apostolos Georgopoulos

**Affiliations:** 1 Core Facility for Microbiology and Hygiene, University Dental Clinic, Vienna, Austria; 2 Medical University of Vienna, Vienna, Austria; 3 The ESCMID Study Group for Legionella Infections (ESGLI), Basel, Switzerland

**Keywords:** dental unit waterlines, disinfection in water systems, *Legionella pneumophila*, microbiological monitoring, *Pseudomonas aeruginosa*

## Abstract

Dental unit waterlines (DUWLs) are highly susceptible to biofilm formation and serve as reservoirs for opportunistic waterborne pathogens, particularly *Legionella pneumophila* and *Pseudomonas aeruginosa*. Effective long-term control of microbial contamination remains challenging due to the complex architecture of DUWLs, intermittent water flow, and the limited applicability of thermal disinfection. Routine microbiological surveillance at the University Dental Clinic Vienna identified recurrent contamination of multiple dental units with *L. pneumophila* and *P. aeruginosa*. To establish an effective and sustainable water management strategy, three disinfection protocols were evaluated under routine clinical conditions: (A) continuous application of electrolytically generated hypochlorous acid (HOCl), maintaining a free chlorine concentration of 0.3–0.6 mg/L, (B) increased HOCl concentration, and (C) HOCl combined with hydrogen peroxide (H_2_O_2_). Water samples were analyzed according to standardized ISO methods for detection of *L. pneumophila*, *P. aeruginosa*, and heterotrophic plate counts (HPC). Protocol A demonstrated reliable balance between microbiological efficacy, technical compatibility, and operational feasibility. Increased HOCl concentrations provided no relevant additional microbiological benefit and were associated with technical impairments, whereas the combined HOCl/H_2_O_2_ protocol showed less microbiological performance. Following standardization to Protocol A, all evaluated units were incorporated into a comprehensive water safety program consisting of continuous disinfection, automated flushing procedures, daily free-chlorine monitoring, and routine microbiological surveillance. During the 3-year follow-up period (2023–2025), sustained suppression of *L. pneumophila* was achieved across the dental unit network, with only isolated residual findings in a single unit. Monitoring further demonstrated that low heterotrophic plate counts did not consistently exclude the presence of opportunistic pathogens, supporting the need for targeted pathogen surveillance in addition to routine HPC monitoring. These findings demonstrate that a standardized HOCl-based water safety program in combination with automated flushing protocols can provide sustainable long-term control of microbial contamination in DUWLs and underscore the importance of pathogen-specific monitoring for a comprehensive microbiological risk assessment.

## 1 Introduction

While severe infections linked to *Legionella spp.* and *Pseudomonas aeruginosa* in dental unit water systems have been reported (1–3), their actual burden is likely underestimated owing to considerable underdiagnosis and limitations in source attribution (4). Consequently, comprehensive water quality surveillance and risk management in dental units remain critical. These measures are essential for protecting both vulnerable patient populations and occupationally exposed dental healthcare workers. The Efforts to control severe infections caused by opportunistic waterborne pathogens, such as *Legionella pneumophila (L. pneumophila)* and *Pseudomonas aeruginosa (P. aeruginosa)*, should prioritize preventive strategies that minimize potential environmental reservoirs, rather than focusing solely on the management of acute clinical disease. *L. pneumophila* has been increasingly recognized as a cause of community-acquired pneumonia, is associated with high morbidity, and represents an important global public health concern (5). *P. aeruginosa* remains one of the leading causes of health care- and ventilator-associated pneumonia and is a prominent respiratory pathogen in intensive care units (6). *P. aeruginosa* is classified by the World Health Organization as a critical-priority pathogen due to its high levels of antimicrobial resistance and limited therapeutic options (7). Although antimicrobial resistance in *Legionella* species remains relatively uncommon, the limited number of effective therapeutic agents underscores the importance of preventive infection control measures. Both pathogens are ubiquitous in natural and engineered water systems and possess a strong capacity to adapt to diverse environmental conditions, including biofilm-associated habitats (8).

Immunocompromised individuals, older patients, and those with chronic underlying conditions are particularly vulnerable when exposed to contaminated water sources or aerosols in health care settings. In hospital settings, thermal disinfection of water systems—also referred to as thermal or pasteurization treatments—is commonly used. This procedure involves temporarily raising water temperatures above 60 °C according to standardized protocols. Aerosol-generating processes, such as those associated with showers, air conditioning systems, or medical equipment, play a key role in the transmission of these pathogens (9). Water systems may serve as a source of *P. aeruginosa* infection in health care settings; however, the exact route of transmission remains unclear (10).

Notably, these opportunistic waterborne pathogens are not restricted to traditional hospital water systems but have also been identified in various other technical water installations, including car wash facilities (11) and, as investigated in this study, dental treatment units (12–15). Due to their complex waterline configurations, frequent use, and aerosol generation during dental procedures, dental unit waterlines (DUWLs) may represent a potential reservoir for microbial contamination. DUWLs typically have a small diameter but a high surface area, and this, combined with an intermittent and slow flow rate contributes to water stagnation (15). This is exacerbated when the entire column of water in the line remains static for prolonged periods, such as overnight or over weekends (13).

According to a meta-analysis by Bayani et al., DUWLs frequently exhibit microbial contamination, with reported prevalence rates of approximately 12% for *Legionella* species and 8% for *Pseudomonas* species (12). Furthermore, several field studies have demonstrated that heterotrophic plate counts (HPC) do not necessarily correlate with the presence of opportunistic pathogens in DUWLs (16). Consequently, reliance on HPC alone may underestimate microbiological risks associated with Legionella spp. and Pseudomonas spp.

In dental treatment units, thermal disinfection is generally not applicable because many system components—such as plastic tubing, seals, and connectors—are not heat-resistant. Therefore, chemical control of water quality is the primary strategy. In this context, disinfectants, such as chlorine-based compounds or ozone, are applied either continuously or intermittently to inhibit microbial growth and inactivate microorganisms. The antimicrobial activity of these agents is based, among other mechanisms, on the oxidation of cellular components, denaturation of proteins, and disruption of bacterial cell membranes (17).

All chemical disinfection strategies share the requirement of achieving sufficient antimicrobial efficacy against waterborne microorganisms while avoiding adverse effects on human skin, mucosal barriers, and the immune system. Additionally, material compatibility is of critical importance, as dental units comprise a wide variety of materials. Structural features, such as junctions, dead spaces, or slightly roughened surfaces, may further promote microbial adhesion and biofilm formation. These factors must therefore be considered when selecting and dosing disinfectants (18). The role of biofilms within water distribution systems and plumbing is recognized as a key factor in the establishment and persistence of chronic colonization by pathogens, such as *L. pneumophila* and *P. aeruginosa*. Biofilms, as well as amoebic host organisms, can protect these pathogens from effective disinfection, including treatment with chlorine-based agents (19). Free-living amoebae may act as environmental reservoirs for Legionella spp., allowing intracellular persistence and enhanced tolerance to routine disinfection procedures (20). Independent of the chemical agent used, regular flushing of water-bearing systems is essential. The primary risk for the proliferation of *Legionella spp.* and *Pseudomonas spp.* lies in lukewarm, stagnant water, such as that found in infrequently used waterlines or during overnight periods (21). Without consistent and standardized flushing protocols, any chemical disinfection strategy is likely to be ineffective over the long term.

Even with appropriate disinfectant concentrations and regular flushing, microorganisms may persist in inaccessible areas or within established biofilms (18, 21). Therefore, continuous microbiological monitoring is essential. Microbiological assessment using membrane filtration in accordance with standardized protocols (22), together with routine verification of disinfectant concentrations, represents a critical component of a comprehensive hygiene management system. Although numerous disinfection systems have been proposed for DUWL management, evidence regarding their long-term effectiveness under routine clinical conditions remains limited. In particular, little information is available regarding the relationship between routine indicator parameters such as HPC and the persistence of opportunistic pathogens during long-term surveillance.

At the University Dental Clinic Vienna, 103 dental treatment units are in daily use. A treatment unit comprises a dental chair, an associated sink, an instrument tray, and multiple water-bearing instruments, the use of which varies depending on the dental specialty.

The water supply is provided by municipal drinking water from Wiener Wasserwerke, which is subject to strict regulatory control and meets high-quality drinking water standards in accordance with Austrian legislation (BGBl. II Nr. 304/2001, as amended) (22). Updated water quality reports are publicly available at: https://www.wien.gv.at/umwelt/trinkwasserqualitaet-aktuelle-werte (23).

In 2021, elevated microbial contamination was detected in the DUWLs of Sirona dental units (Dentsply Sirona (Charlotte, NC, USA) at the University Dental Clinic, Vienna. Such contamination is well documented in the literature (15, 18). While a centralized automated flushing and dosing system had already been established for Planmeca dental units (Planmeca Oy, Helsinki, Finland) in other areas of the clinic, no comparable experience was available for Sirona systems at that time. The existing central system was based on the continuous dosing of hypochlorite-containing disinfectant solutions, which had proven effective in controlling microbial contamination in Planmeca units. Conversely, for Sirona dental units, manufacturer recommendations primarily rely on hydrogen peroxide (H_2_O_2_) cartridge-based systems, which limits the direct transferability of the established hygiene protocol. Against this background, an evaluation of different flushing and disinfection protocols became necessary. The following approaches were examined: (A) application of the standard hypochlorite solution used in the central dosing system, (B) increased hypochlorite concentrations to compensate for potential reduced efficacy in Sirona units, and (C) a combined protocol consisting of standard hypochlorite dosing supplemented with H_2_O_2_ cartridge systems in accordance with the manufacturer's recommendations. The aim was to identify an effective and practically feasible strategy for reducing microbial contamination in Sirona dental units. All procedures and evaluations were conducted under the supervision of a clinical microbiologist and infection control specialists, ensuring valid microbiological assessment and adherence to established hygiene standards.

In addition to microbiological efficacy, economic and environmental aspects were considered. In particular, the use of H_2_O_2_ cartridge systems is associated with increased resource consumption, as continuous replenishment is required and additional packaging waste is generated. This may negatively affect both operational costs and environmental sustainability, and therefore represents an important factor in selecting an optimal hygiene strategy. Furthermore, continuous or automated disinfection approaches have been shown to be more effective than intermittent procedures in maintaining microbiological water quality in dental unit systems.

Based on these considerations, the implementation of an automated flushing and disinfection system in dental units manufactured by Dentsply Sirona was evaluated in combination with different chemical disinfection strategies.

## 2 Materials and methods

2.1 This study was conducted as a prospective, comparative evaluation under routine clinical conditions in a dental health care setting. The primary aim was to assess the effectiveness of different disinfection strategies for controlling microbial contamination in DUWLs. All procedures were performed under the continuous supervision of a clinical microbiologist and a hospital infection control specialist to ensure adherence to established hygiene standards and valid microbiological assessments. When contamination was identified, immediate professional biofilm removal measures were implemented.

### 2.2 Ethical statement

The study exclusively analysed environmental water samples. No patient-related data, human specimens, or identifiable information were collected or processed. Therefore, ethical approval was not required.

### 2.3 Dental units and disinfection protocols

For the evaluation of three different water treatment protocols (A, B, and C), a total of nine dental units were selected in the second unit of the dental clinic at the Medical University of Vienna. The SAFEWATER® system generates a hypochlorous acid (HOCl) stock solution from water, salt, and electricity. The target free chlorine concentration was maintained within a range of 0.3–0.6 mg/L. Minor fluctuations within this range are expected in drinking water systems and may result from hydraulic conditions, water consumption patterns, residence times, incomplete mixing, and chlorine losses caused by turbulence and degassing. Routine photometric monitoring was therefore performed to verify compliance with the intended operational range by the in-house-technician-team.

**Figure 1:**
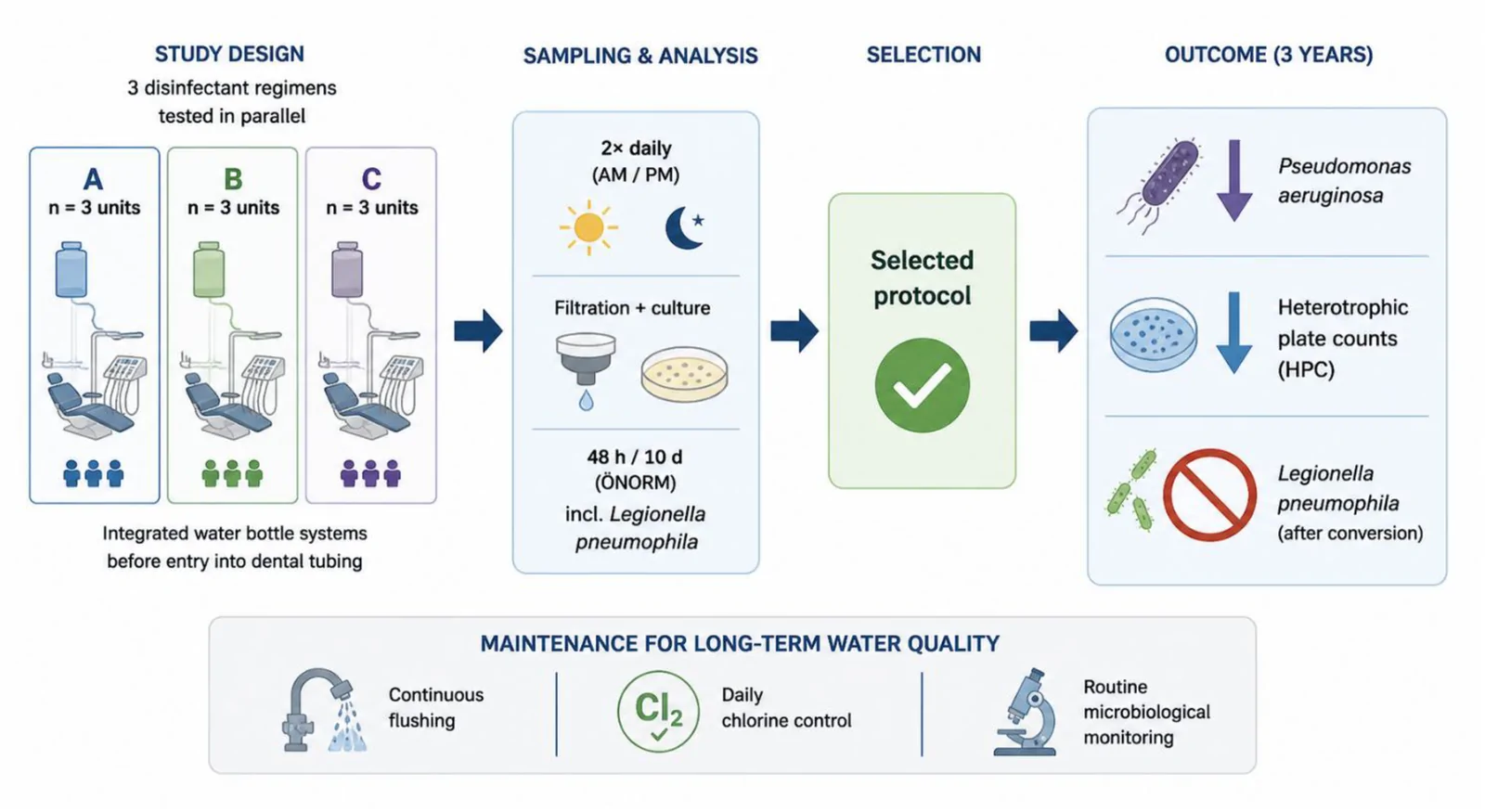
Graphical abstract (AI-assisted content generation: ChatGPT (OpenAI) was used to support the design and wording of the graphical abstract. Final content and scientific accuracy were reviewed and approved by the authors.

The nine units were equally distributed along the water supply system and operated with three disinfection strategies: (A) standard Blue Safety (Blue Safety GmbH, Münster, Germany) using electrolytically generated hypochlorous acid (HOCl), maintaining a target free chlorine concentration of 0.3–0.6 mg/L (equivalent to approximately 0.3–0.6 ppm), (B) Blue Safety protocol with increased hypochlorous acid concentration (0.3–0.6 mg/L + 0.18 mg/L), and (C) Blue Safety protocol supplemented with 1.41% hydrogen peroxide (H_2_O_2_) in the water tanks of dental units (Figure 1).

### 2.4 Sampling

Water sampling for evaluation was performed during two evaluation periods in 2022 (May/June and September) to assess protocol stability under routine clinical conditions, including elevated ambient temperatures and a 2-week clinical shutdown associated with water stagnation. Fourteen sampling series were conducted, with samples collected twice daily (morning and afternoon) to evaluate potential effects of overnight stagnation, resulting in a total of 28 sampling events.

For each sampling event, 250 mL water samples were collected from selected dental unit outlets (second and fourth instrument lines and the air–water syringe), which were considered particularly susceptible to biofilm formation due to lower usage frequency. Prior to sampling, all instruments were removed and disinfected. Samples were transported immediately to the laboratory, where residual disinfectants were neutralized using sodium thiosulfate for hypochlorous acid and catalase for hydrogen peroxide.

Sampling and microbiological analyses according to the relevant ISO standards, including ISO 19458 (sampling for microbiological analysis), ISO 11731 and ISO 11731-2 (detection and enumeration of *Legionella pneumophila*), ISO 16266 (detection and enumeration of *Pseudomonas aeruginosa*), ISO 6222 (heterotrophic plate counts), and ISO 7393-2 (determination of free chlorine concentrations). All analyses were in accordance with internal standard operating procedures (SOPs) and laboratory quality management procedures.

For microbiological investigations membrane filtration techniques was applied. For detection of *P. aeruginosa*, 100 mL water samples were filtered through nitrocellulose membranes (MIL-HABG047S6, Merck KGaA, Darmstadt, Germany) and cultured on Pseudomonas CFC Agar (9145 Biosolute, Th. Geyer GmbH & Co. KG, Renningen, Germany) at 37 °C for 48 h.

For detection of *L. pneumophila*, 100 and 20 mL aliquots were filtered, treated with acid buffer (9994 Biosolute, Th. Geyer GmbH & Co. KG, Renningen, Germany), and cultured on GVPC agar (9660 Biosolute, Th. Geyer GmbH & Co. KG, Renningen, Germany) at 37 °C for up to 10 days.

Heterotrophic plate counts (HPC) at 22 °C and 37 °C were determined according to ISO 6222 by inoculation of 1 mL water samples onto yeast agar (9688 Biosolute, Th. Geyer GmbH & Co. KG, Renningen, Germany).

Repeated sampling at predefined intervals throughout the study period served as a measure of reproducibility and temporal stability. Each sample represented an independent environmental measurement and no technical replicate cultures were performed.

Where microbiological values exceeded predefined action thresholds [L. pneumophila = 0 CFU/100 mL, P. aeruginosa = 0 CFU/100 mL, and heterotrophic plate counts (HPC) at 22 °C and 37 °C < 100 CFU/mL], corrective measures were initiated according to the established hygiene protocol under the supervision of the clinical microbiologist and chief hygienist. These measures included intensified flushing, adjustment of HOCl dosing, repeat microbiological sampling, and professional biofilm-removal procedures where necessary.

Statistical analyses were performed to compare microbiological outcomes among the three disinfection protocols during the protocol evaluation phase. Although only three dental units were assigned to each protocol, the analysis was based on repeated environmental measurements obtained during 14 microbiological assessments conducted between April and July 2022, including both morning and afternoon sampling. Consequently, the statistical evaluation was performed on repeated observations rather than on a single measurement per unit. Given the limited number of dental units per protocol, the results should be interpreted as exploratory. Statistical analysis was performed using analysis of variance (ANOVA) to assess differences between disinfection protocols and sampling times (morning vs. afternoon). Statistical significance was defined as *p* < 0.05, and significant findings were further explored using *post hoc* pairwise comparisons (Supplementary B).

### 2.5 Automated water treatment and flushing

HOCl was administered via an external automated water treatment system (BLUE SAFETY GmbH Medical technology manufacturer in Münster, Germany), which enabled continuous or interval-based dosing. Flushing procedures included an initial flushing at the beginning of the working day (2–3 min per waterline), intermittent flushing between patients (20–30 s), and automated flushing outside operating hours (at least twice daily). The units were supplied with municipal drinking water. Free chlorine concentrations (ppm) were measured on working days using a photometric test and documented electronically as part of the routine quality assurance program.

### 2.6 Postintervention monitoring

After completion of the evaluation phase, all included dental units were transitioned to Protocol A (standard HOCl 0.3–0.6 mg/L) since this shows to sufficient effective without harming equipment. To assess long-term water quality, continuous microbiological monitoring was conducted from 2023 to 2025. The results were evaluated in accordance with above named hygiene guidelines and standard operating procedures.

### 2.7 Quality assurance and outcome measures

All microbiological analyses according to the respective ISO standards and internal laboratory standard operating procedures under the supervision of a clinical microbiologist. Culture media quality control upon receipt of each new batch using appropriate reference strains and sterility controls in accordance with laboratory quality management procedures and manufacturer recommendations. Disinfectant neutralization was carried out using sodium thiosulfate and catalase prior to cultivation as required by the applicable ISO standards (22).

Repeated sampling at predefined intervals throughout the study period served as a measure of reproducibility and temporal stability.

Primary outcome measures were the detection and enumeration of *Legionella pneumophila* and *Pseudomonas aeruginosa* according to ISO 11731 and ISO 16266. Secondary outcome measures included heterotrophic plate counts (HPC) at 22 °C and 37 °C according to ISO 6222, comparison of microbiological reduction between disinfection protocols, and long-term microbiological stability following implementation of the selected water management strategy. Results were interpreted according to the corresponding ISO standards, applicable hygiene guidelines, and predefined action thresholds.

## 3 Results

### 3.1 Baseline microbiological contamination

Baseline surveillance revealed substantial microbiological contamination across the investigated dental units. Repeated detections of *Legionella pneumophila* and *Pseudomonas aeruginosa* were observed in several units prior to implementation of the optimized water management strategy. The extent of contamination varied between units, with some units demonstrating repeated pathogen detection, whereas others were primarily characterized by elevated heterotrophic plate counts (HPC). A notable finding was the inconsistent relationship between HPC values and pathogen detection. Several units exhibited *L. pneumophila* counts exceeding action thresholds despite low HPC values, indicating that routine heterotrophic bacterial counts did not consistently reflect the presence of opportunistic pathogens.

**Figure 2:**
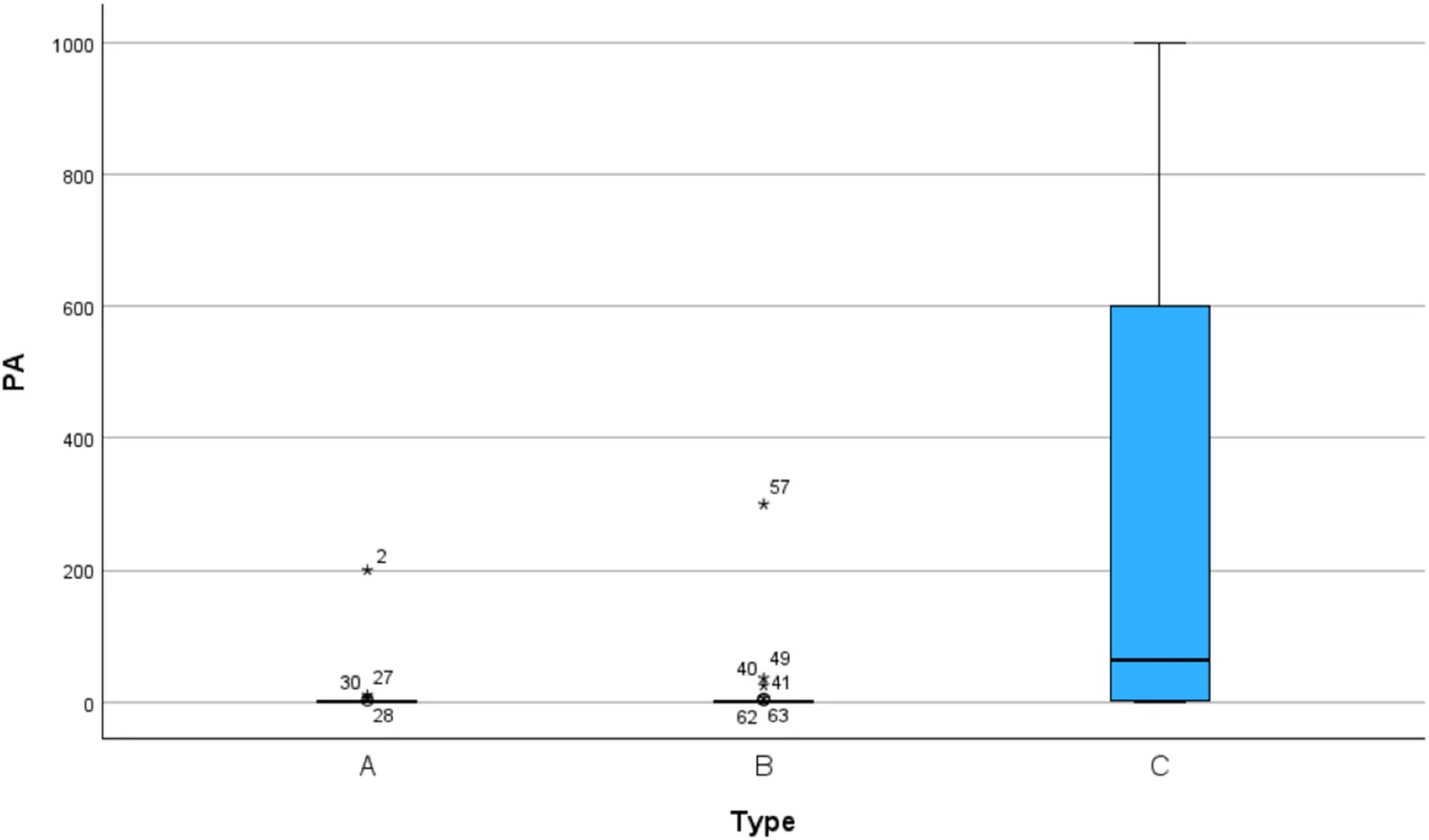
Evaluation for P. aeruginosa: Protocol A show highest bacterial reduction, Protocol B similar and Protocol C show high bacterial contamination with over 100 CFU.

**Figure 3:**
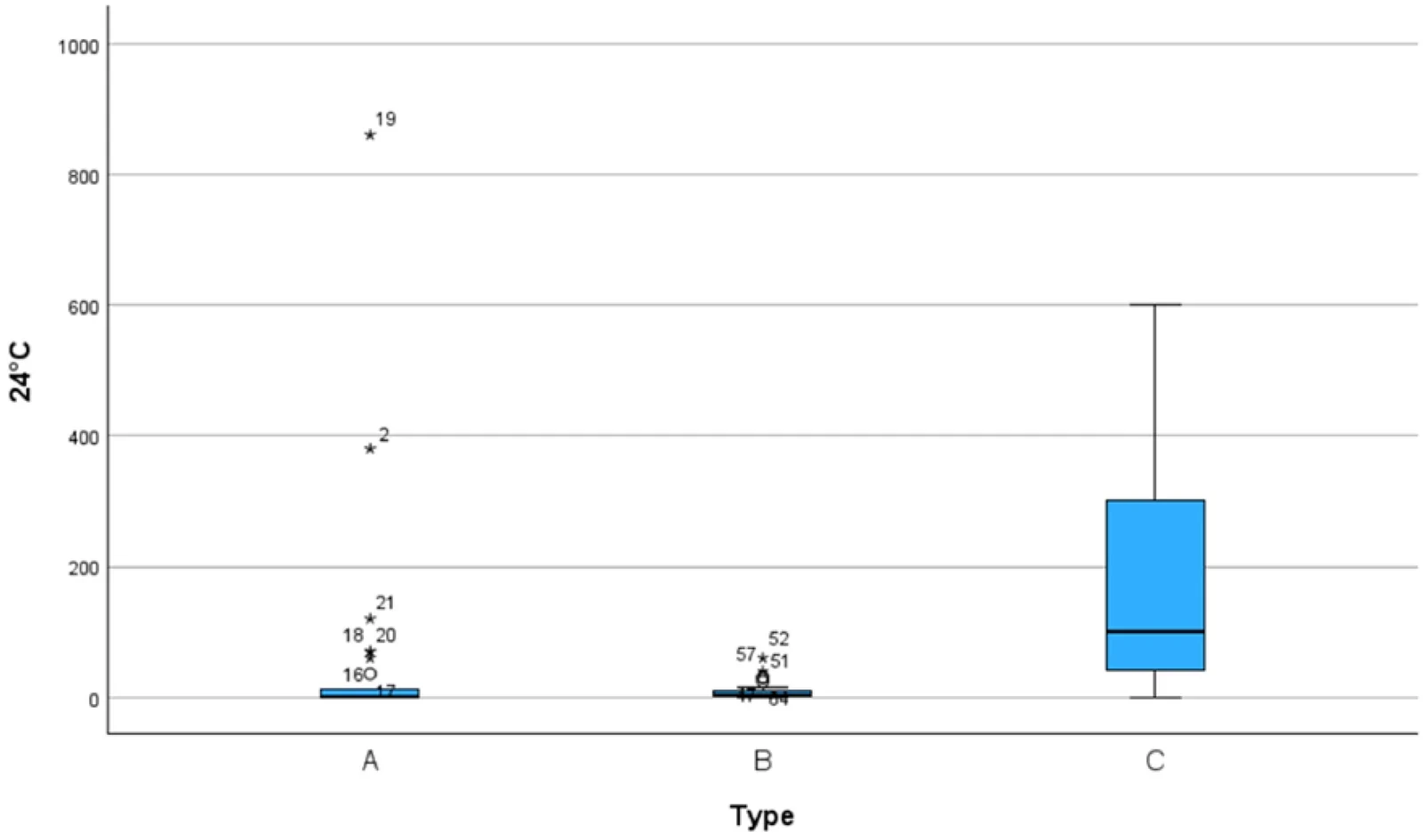
Evaluation heterotrophic bacterial count: protocol A show highest bacterial reduction, protocol B similar and protocol C show high bacterial contamination with over 100 CFU.

**Figure 4:**
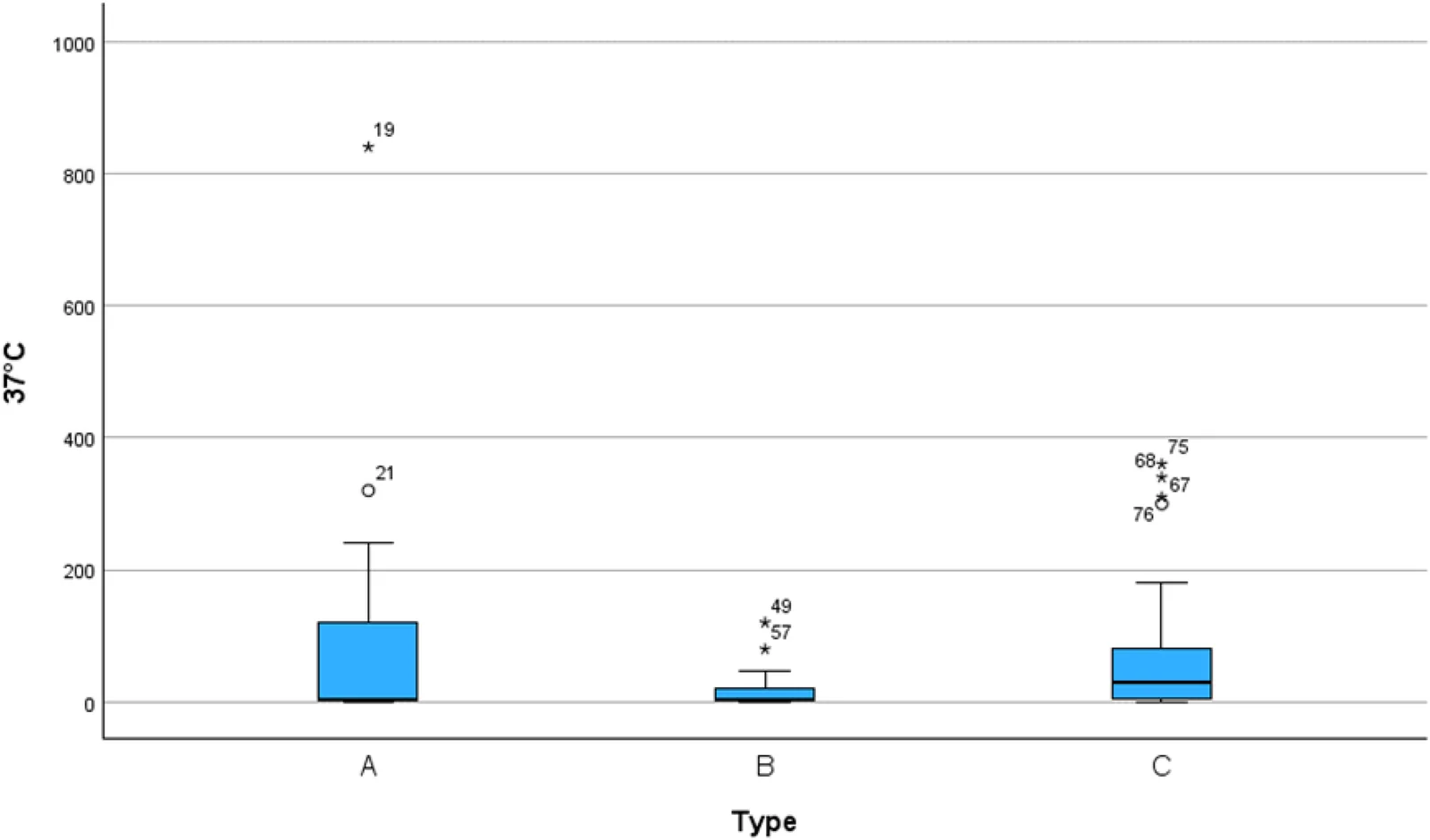
Evaluation heterotrophic bacterial count: Protocol A and C show highest bacterial count with over 100 CFU. Protocol B minor contamination.

**Figure 5:**
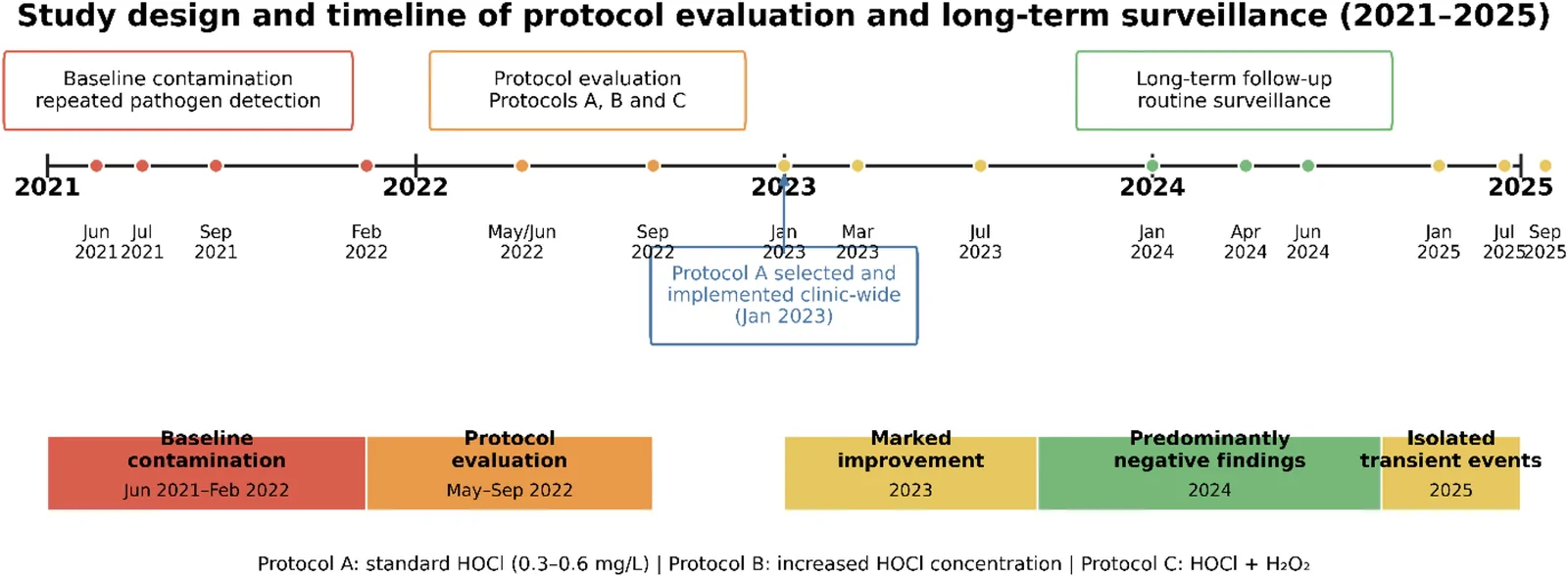
Timeline of disinfection protocol evaluation and long-term microbiological surveillance of dental unit waterlines (DUWLs). The heatmap summarizes the overall microbiological status during each study phase, demonstrating progressive reduction of contamination and sustained microbiological stability during long-term follow-up (2023–2025), with only sporadic transient increases in heterotrophic plate counts (HPC) and occasional detections of P. aeruginosa.

**Figure 6:**
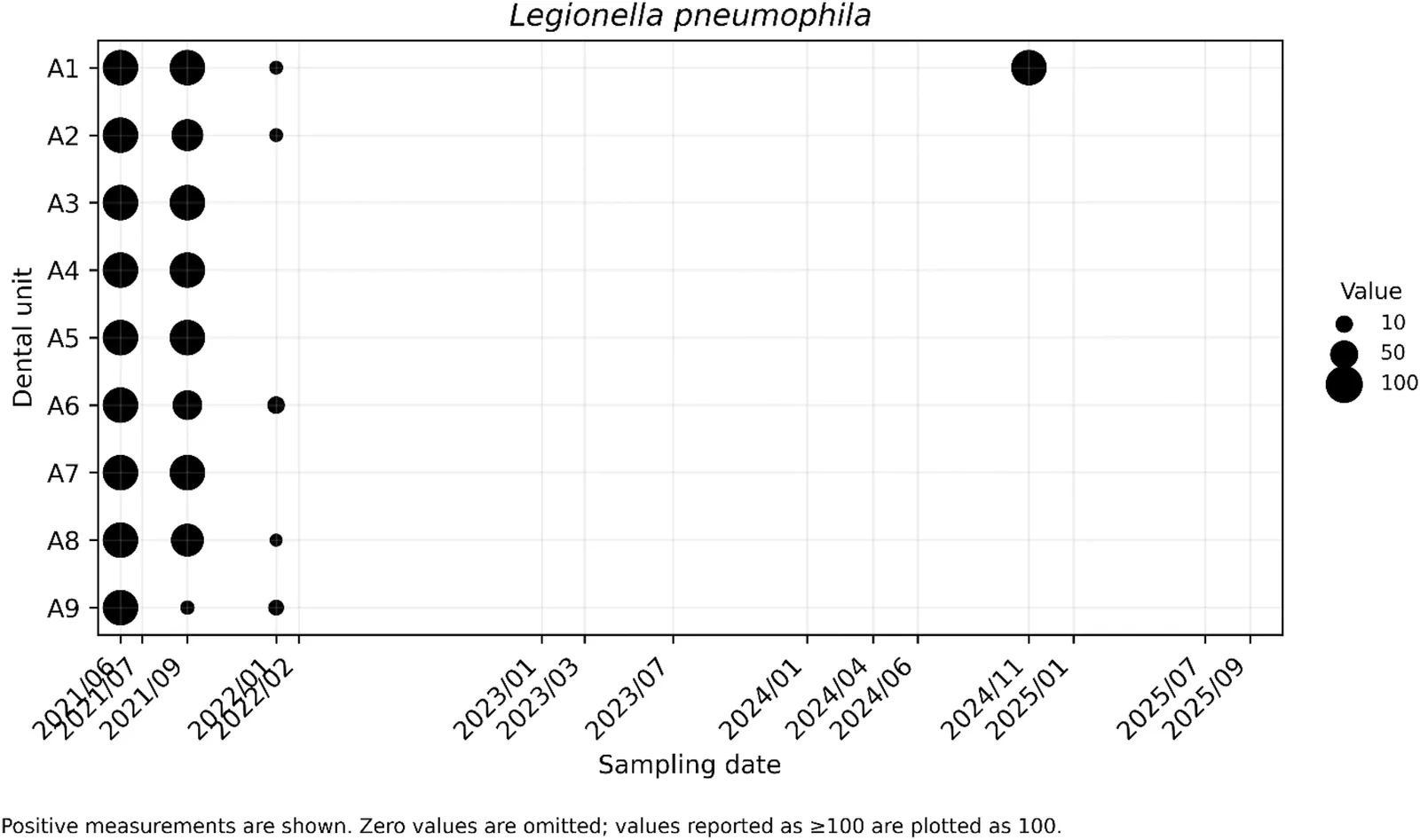
Longitudinal detection of Legionella pneumophila in dental unit waterlines.

**Figure 7:**
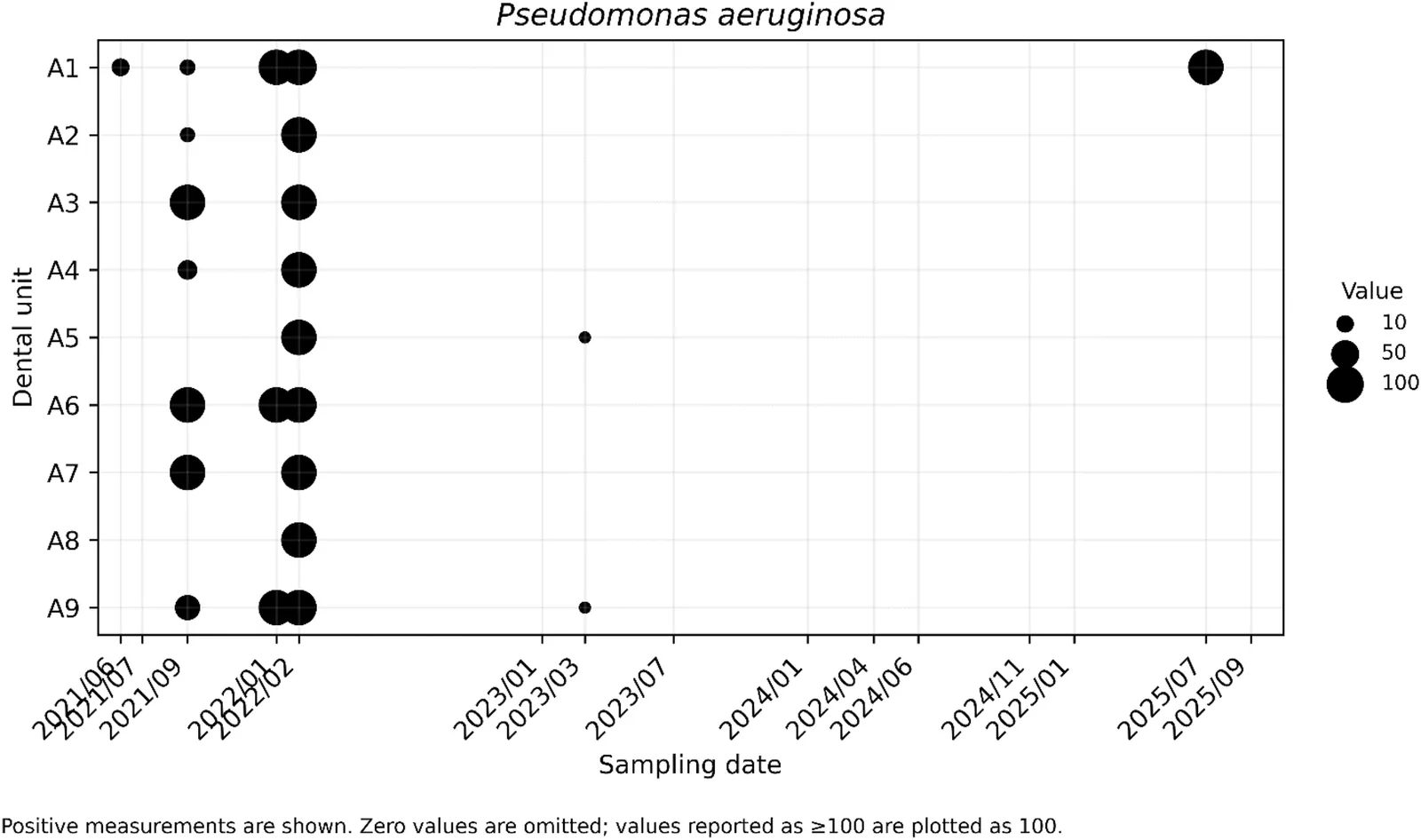
Longitudinal detection of Pseudomonas aeruginosa in dental unit waterlines.

**Figure 8:**
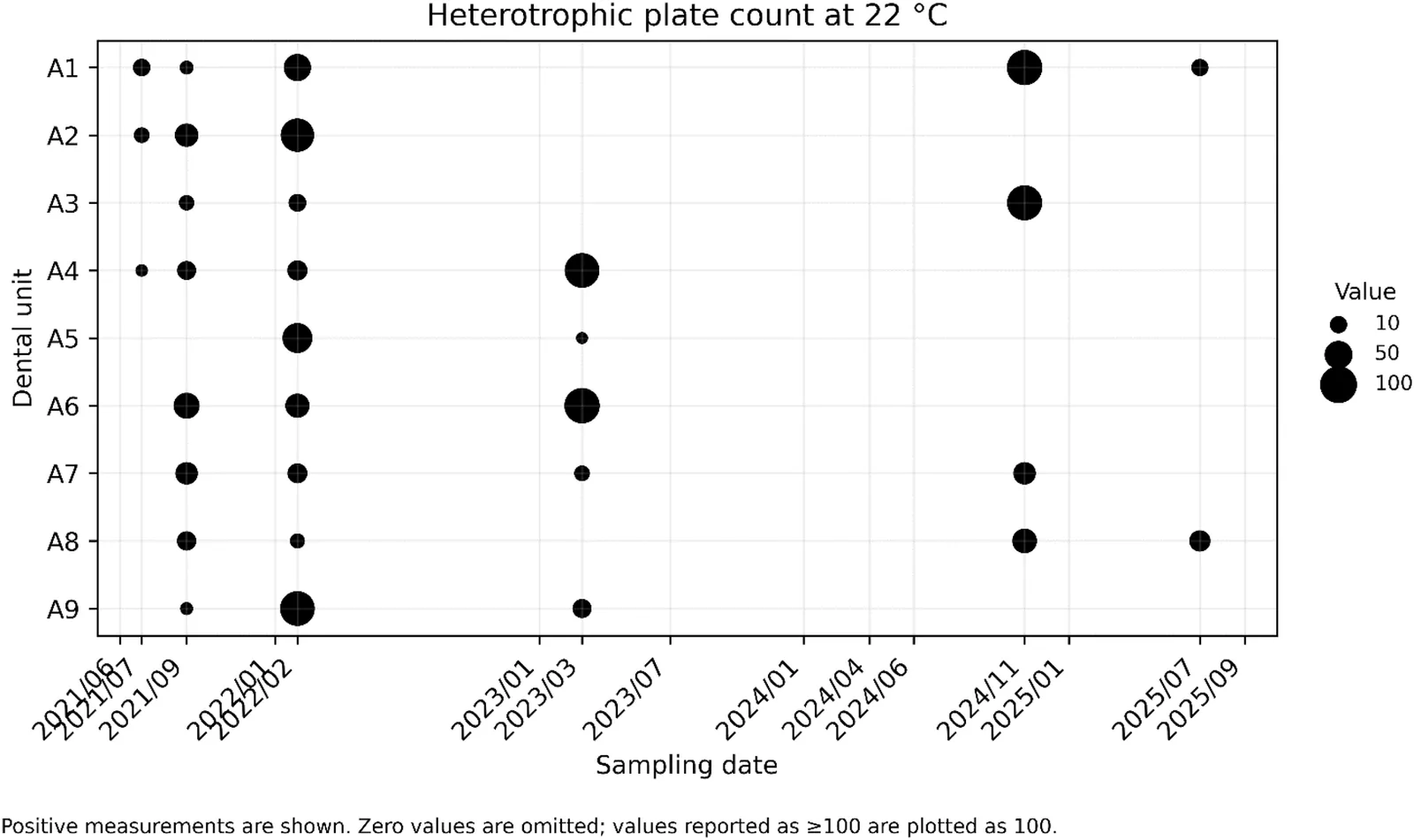
Longitudinal heterotrophic plate counts at 22 °C in dental unit waterlines.

**Figure 9:**
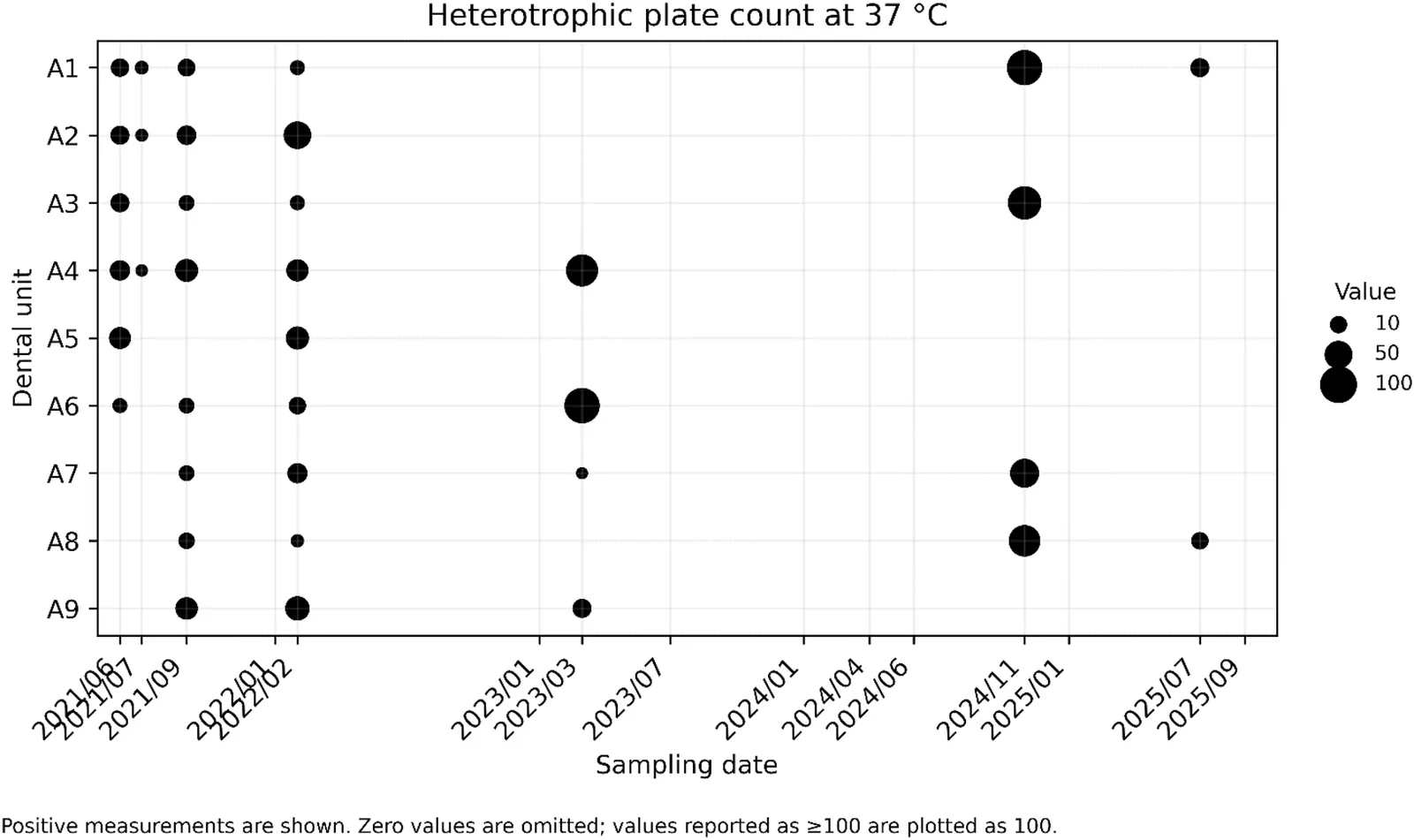
Longitudinal heterotrophic plate counts at 37 °C in dental unit waterlines.

Protocol A demonstrated the best overall performance in terms of antimicrobial reduction and material safety. It was therefore selected for long-term implementation (Figure 2). Following implementation of the disinfection protocols, a marked reduction in microbiological contamination was observed across all units (example Figures 3–9).

Detailed unit-specific microbiological data and longitudinal surveillance results are provided in Supplementary A, Figures S1–S9.

### 3.2 Comparative evaluation of disinfection protocols

Three disinfection protocols were evaluated under routine clinical conditions.

During the protocol evaluation phase, *Legionella pneumophila* was not detected in any water sample following application of protocols A, B, or C, preventing statistical comparison between the disinfection strategies for this parameter.

For *Pseudomonas aeruginosa*, markable differences were observed among the evaluated protocols (overall ANOVA, *p* < 0.001) (Figure 2). *Post hoc* analysis demonstrated no significant difference between Protocol A (standard HOCl concentration) and Protocol B (increased HOCl concentration) (*p* = 0.995). In contrast, Protocol C (HOCl combined with H_2_O_2_) performed significantly worse than both Protocol A (*p* < 0.001) and Protocol B (*p* < 0.001), indicating reduced effectiveness in controlling *P. aeruginosa* contamination.

Similar findings were observed for heterotrophic plate counts (HPC) at 24 °C (Figure 3). No significant difference was found between Protocols A and B (*p* = 0.419), whereas Protocol C showed markable higher bacterial counts compared with both Protocol A (*p* = 0.003) and Protocol B (*p* < 0.001). For HPC at 37 °C (Figure 4), no statistically significant differences were observed between any of the protocols (A vs. B: *p* = 0.098; A vs. C: *p* = 0.993; B vs. C: *p* = 0.124) (Supplementary B, Tables S1, S2 and Supplementary B, Figures S10–S13).

Overall, Protocol A achieved microbiological control comparable to Protocol B despite the lower HOCl concentration, whereas Protocol C exhibited the lowest overall effectiveness. Given the absence of additional microbiological benefit from increased HOCl dosing and the occurrence of technical impairments in dental unit components during Protocol B, Protocol A was selected as the standardized water treatment protocol for long-term implementation.

*L. pneumophila* was not detected following any of the evaluated disinfection protocols during the protocol evaluation phase.

Although Protocol B achieved microbiological results comparable to Protocol A, the higher HOCl concentration was associated with material-related impairments in some dental units. Observed effects included damage to rubber seals and crack formation in plastic tubing components, which required technical intervention. Based on these observations, Protocol A was selected for clinic-wide implementation (Supplementary B, Tables S1, S2 and Supplementary B, Figures S10–S13).

### 3.3 Long-term surveillance following implementation of Protocol A

Following standardization to Protocol A, all evaluated units were incorporated into a structured water safety program including continuous disinfection, automated flushing procedures, daily monitoring of free chlorine concentrations, and routine microbiological surveillance.

Longitudinal surveillance of nine dental units (Unit 1–9) was conducted between June 2021 and September 2025. A comparison of the contamination per unit before and after the HOCl implementation is provided in Table 1.

**Table 1:** Longitudinal microbiological outcomes in dental unit waterlines before and after implementation of the standardized HOCl-based protocol.

Outcome	Baseline contamination phase (2021–2022)	Long-term follow-up (2023–2025)
Dental units positive for *L. pneumophila*	9/9 units showed at least one positive sample	1/9 units during routine surveillance
Dental units positive for *P. aeruginosa*	8/9 units showed at least one positive sample	3/9transient positive event
Dental units with elevated HPC (22 °C and/or 37 °C)	Frequent elevations observed across multiple units	Occasional transient elevations in individual units; no persistent increases detected
Units with repeated microbiological deviations	9/9 units	1/9 units (Unit1)
Units with stable microbiological control	0/9 units	8/9 units

The temporal distribution of positive findings for *Legionella pneumophila*, *Pseudomonas aeruginosa*, heterotrophic plate counts (HPC) at 22 °C, and HPC at 37 °C is shown in Figures 3–7.

During the baseline contamination phase (2021–2022), positive findings were frequently detected for all microbiological parameters. *L. pneumophila* was detected in 25 of 45 samples (55.6%), *P. aeruginosa* in 20 of 45 samples (44.4%), while positive HPC findings were observed in 26 of 45 samples (57.8%) at 37 °C and 20 of 45 samples (44.4%) at 22 °C.

Following implementation of the standardized HOCl-based protocol, the frequency of positive findings decreased markedly. Only a single *L. pneumophila* detection was recorded during the 3-year follow-up period (1/90 samples, 1.1%), representing an isolated event in November 2024. *P. aeruginosa* was detected in only 3 of 90 samples (3.3%). Positive HPC findings were reduced to 10 of 90 samples (11.1%) at 37 °C and 11 of 90 samples (12.2%) at 22 °C. Overall, 89 of 90 samples (98.9%) remained negative for *L. pneumophila*, 87 of 90 samples (96.7%) remained negative for *P. aeruginosa*, while 80 of 90 samples (88.9%) and 79 of 90 samples (87.8%) remained negative for HPC at 37 °C and 22 °C, respectively (Table 2).

**Table 2:** Comparison of microbiological findings before and after implementation of the standardized HOCl-based protocol.

Outcome	Baseline contamination phase (2021–2022) (*n* = 45)	Long-term follow-up (2023–2025) (*n* = 90)
*Legionella pneumophila*	25 positive (55.6%); 20 negative (44.4%)	1 positive (1.1%); 89 negative (98.9%)
*Pseudomonas aeruginosa*	20 positive (44.4%); 25 negative (55.6%)	3 positive (3.3%); 87 negative (96.7%)
HPC 37 °C	26 positive (57.8%); 19 negative (42.2%)	10 positive (11.1%); 80 negative (88.9%)
HPC 22 °C	20 positive (44.4%); 25 negative (55.6%)	11 positive (12.2%); 79 negative (87.8%)

At baseline (June 2021), contamination with *L. pneumophila* was detected in all dental units (Figure 6), with concentrations reaching or exceeding 0 CFU/100 mL in most units. During the protocol evaluation phase, contamination levels decreased. Following implementation of the standardized HOCl-based protocol in January 2023 which includes flushing, dosing and monitoring, *L. pneumophila* remained undetectable in nearly all units throughout the remainder of the surveillance period. Only one isolated positive finding was observed, most notably in unit 1 in November 2024.

A comparable pattern was observed for *P. aeruginosa* (Figure 7). Positive findings were common during the baseline phase and several measurements exceeded 0 CFU/100 mL. During and after the protocol evaluation period, the frequency and magnitude of positive findings decreased markedly. From 2023 onwards, *P. aeruginosa* was largely absent from the dental unit waterlines, with only sporadic detections in individual units. The most pronounced late recurrence was observed in unit 1 in July 2025.

Heterotrophic plate counts at both 22 °C and 37 °C showed substantial variability during the baseline period and the protocol evaluation phase (Figures 8, 9). Elevated counts exceeding the target value of 100 CFU/mL were observed in several units, particularly during 2021 and early 2022. Following introduction of the, standardized HOCl-based protocol HPC values decreased considerably and remained low in most dental units.

Despite the overall improvement, intermittent increases in HPC were observed in a limited number of units. In November 2024, elevated HPC values were detected in units 1, 3, 7, and 8, whereas *L. pneumophila* and *P. aeruginosa* remained absent in most units. No sustained recolonization pattern was observed after implementation of the standardized protocol (timeline for each single unit available in Supplementary Figures S1–S9).

- Overall, the surveillance data demonstrate a substantial reduction in microbiological contamination during the protocol evaluation phase and sustained microbiological control after implementation of the standardized HOCl-based protocol from January 2023 onwards.

## 4 Discussion

### 4.1 Effectiveness of the HOCl-based water safety program

Following the implementation of the optimized water management strategy in Jan. 2023, by continuous application of electrolytically generated hypochlorous acid (HOCl), maintaining a free chlorine concentration of 0.3–0.6 mg/L and regular flushing, a marked reduction in microbiological contamination was observed in all units, except unit 1. The present evaluation of different disinfection protocols revealed that increasing the concentration of hypochlorite did not provide a meaningful additional microbiological benefit while simultaneously introducing technical disadvantages that affected system components. Oxidizing disinfectants used in DUWL systems may adversely affect technical components over time. O'Donnell et al. reported intermittent failures of DUWL disinfection systems attributable not only to operator error but also to corrosion-related blockage of disinfectant intake valves, indicating that chemical disinfectants may contribute to equipment degradation during long-term use (18). In this study, technical impairments were observed when higher disinfectant concentrations were applied, which is consistent with these reports. After completion of the evaluation phase, all dental units were standardized to an HOCl-based protocol (0.3–0.6 mg/L). Since the implementation of this standard protocol in 2023, no equipment failures attributable to material degradation or disinfectant-related damage have been observed. Additionally, the disinfectant concentration was monitored daily using a Hanna® photometric test for free chlorine (reference range 0.3–0.6 ppm or mL/L), enabling immediate detection of deviations and timely corrective action. The comparatively lower effectiveness observed for the combined disinfection protocol C may be explained by the known chemical interaction between H_2_O_2_ and HOCl. Reaction kinetic studies have shown that H_2_O_2_ reacts rapidly with HOCl, resulting in the consumption of free oxidizing species and a potential reduction in the availability of HOCl as an antimicrobial agent (24) mechanistic studies have similarly described the rapid redox reaction between these compounds, supporting the concept that their simultaneous presence may alter antimicrobial activity (25). From a practical perspective, several authors have suggested that oxidizing disinfectants such as HOCl and H_2_O_2_ may be more effective when applied sequentially or in separate treatment steps rather than in combination, as direct interaction between the compounds may partially reduce their oxidizing capacity (24, 25). Although the findings do not allow for a definitive conclusion regarding direct chemical antagonism under clinical DUWL conditions, they are consistent with the known reactivity of these oxidants and support the hypothesis that their simultaneous application may reduce the effective concentration of active disinfectant species.

### 4.2 Long-term control of Legionella and Pseudomonas

The long-term control of opportunistic, waterborne pathogens remains one of the top challenges in drinking water management. Tuvo et al., for example, observed renewed Legionella contamination within a few weeks of a shock treatment with hydrogen peroxide, necessitating repeated interventions and additional control measures (26). Similarly, several authors have highlighted the persistence of biofilm-associated microorganisms as a major obstacle to sustainable decontamination of DUWL systems (12, 13, 18, 20). In the present study, with one exception, no *L. pneumophila* was detected following implementation of the standardized HOCl-based protocol, and during the 3-year follow-up period, only sporadic, minor detections of *P. aeruginosa* occurred. These results suggest that the chosen water management strategy enabled sustained microbiological control beyond the initial intervention phase.

An interesting finding of the present study was the inconsistent relationship between heterotrophic plate counts (HPC) and the detection of opportunistic pathogens. Several baseline samples yielded *L. pneumophila* counts ≥100 CFU/100 mL despite low HPC values, in some cases approaching zero. These observations indicate that routine HPC measurements alone may not reliably reflect the microbiological risks associated with DUWL contamination. Similar findings have been reported previously. Arvand and Hack detected *Legionella* spp. in 27.8% of dental units, whereas elevated heterotrophic bacterial counts were observed in only 17% of samples, demonstrating that pathogen occurrence cannot necessarily be inferred from overall bacterial counts alone (16). Consequently, several authors have emphasized that HPC should primarily be regarded as an operational indicator of biofilm activity and system performance rather than a direct surrogate marker for the presence of opportunistic pathogens (20). A possible explanation for this discrepancy lies in the biofilm-associated ecology of DUWLs. Biofilms provide physical protection against disinfectants and may facilitate the persistence of microorganisms within ecological niches (27) that are not adequately represented by routine HPC measurements. In particular, free-living amoebae have been described as environmental reservoirs for *Legionella* spp., allowing intracellular survival and increased tolerance to routine disinfection procedures (20). Under such conditions, reductions in the general heterotrophic background flora may occur without complete elimination of biofilm-associated opportunistic pathogens. Furthermore, recent molecular studies have demonstrated that conventional culture-based methods may underestimate the true microbial diversity present within DUWL biofilms. Ditommaso et al. detected *Legionella* DNA in virtually all investigated DUWL samples using PMA-qPCR, whereas only a small proportion of samples were culture-positive (20). Although culture-based methods remain essential for regulatory compliance and risk assessment, these findings further support the concept that microbiological surveillance based solely on HPC may underestimate the presence of clinically relevant microorganisms. Beyond patient safety, sustained control of DUWL contamination is also relevant for occupational health. Although the overall epidemiological risk for dental personnel appears to be low, repeated exposure to contaminated aerosols has been associated with increased seropositivity to *Legionella* spp. in some investigations, highlighting the importance of continuous microbiological surveillance and preventive water management measures (28).

The implementation of a standardized automated flushing and disinfection system based on HOCl resulted in a marked and sustained stabilization of water quality. Subsequent long-term monitoring revealed predominantly negative microbiological findings, with only occasional contamination events that resolved rapidly upon follow-up testing. The advantages of such standardized automated flushing and disinfection systems have previously been described by O'Donnell et al. in the context of their application in Planmeca units. Comparable benefits were observed in the present study for units manufactured by Dentsply Sirona. Additionally, manual flushing at the beginning and end of each working day, as well as between patients, proved to be an effective adjunctive measure for reducing microbial load in DUWL systems. The sustained microbiological stability observed during the long-term follow-up period should not be attributed to HOCl dosing alone. Rather, it reflects the combined effect of a standardized waterline management strategy, including continuous HOCl application, routine flushing procedures, regular microbiological surveillance, and timely corrective actions when deviations were detected. In addition, staff compliance and active on-site hygiene management played an important role in maintaining long-term system performance by ensuring adherence to protocols and facilitating rapid responses to technical or microbiological irregularities.

### 4.3 Limitations

Several limitations should be considered when interpreting the findings of this study. First, the investigation was conducted at a single university dental clinic, which may limit the generalizability of the results to other clinical settings with different DUWL configurations, water supplies, or maintenance procedures. Second, microbiological assessment was based on culture-dependent methods in accordance with current ISO standards. While these methods remain the reference standard for regulatory compliance and risk assessment, they may underestimate the total microbial diversity present within DUWL biofilms. Furthermore, the study was not designed to characterize biofilm composition, microbial community dynamics, or potential changes in disinfectant tolerance over time. Molecular approaches such as qPCR, 16S rRNA sequencing, or metagenomic analyses were not performed and therefore potential ecological shifts within the microbial community could not be assessed. The extent of biofilm formation within individual dental unit waterlines was not directly quantified and therefore could not be standardized between units. However, repeated professional biofilm-removal procedures had been performed by a specialized service provider whenever elevated microbiological findings were detected prior to the evaluation phase. Despite these measures, differences in residual biofilm burden between units cannot be excluded and may have contributed to variability in microbiological outcomes. Nevertheless, the sustained microbiological stability observed during the 3-year follow-up period suggests that the implemented management strategy remained effective under routine clinical conditions.

The protocol evaluation included only three dental units per intervention group, which may limit the generalizability of the results. Although repeated microbiological assessments were performed throughout the evaluation period, the statistical comparisons should be regarded as exploratory. In contrast, the long-term surveillance data were collected to assess real-world performance of the implemented protocol and are therefore presented descriptively rather than as inferential statistical evidence.

Detailed information regarding the utilization rate of individual dental units was not available, as access to clinical scheduling and patient-related data was restricted for legal and data protection reasons. To account for potential effects of daily operation and water stagnation, samples during the evaluation phase were collected 2 times daily, in the morning and the afternoon. No significant differences were observed between morning and afternoon samples. Nevertheless, variations in utilization between individual units cannot be excluded and may have contributed to variability in microbiological findings.

Despite these limitations, the study provides valuable real-world data on the implementation and long-term performance of a standardized HOCl-based water safety program under routine clinical conditions. The 3-year surveillance period represents a particular strength, allowing assessment not only of immediate disinfection efficacy but also of the long-term sustainability of microbiological control measures.

## 5 Conclusion

The findings of this study demonstrate that sustainable control of *Legionella pneumophila* and *Pseudomonas aeruginosa* in dental unit waterlines requires more than chemical disinfection alone. Long-term microbiological stability was achieved through an integrated water safety program combining continuous HOCl-based disinfection, routine flushing procedures, regular microbiological surveillance, and consistent adherence to standardized operating procedures, thereby supporting both patient safety and occupational health in dental practice.

## Data Availability

The original contributions presented in the study are included in the article/Supplementary Material, further inquiries can be directed to the corresponding author.
