# Supplementary material for: Long-term control of Legionella pneumophila and Pseudomonas aeruginosa in dental unit waterlines following optimization of a hypochlorous acid-based water safety program
Source: Front Oral Health. 2026 Aug 11;7:1903381. doi: 10.3389/froh.2026.1903381 (PMC13503258; doi:10.3389/froh.2026.1903381)
Supplement: Supplementary file 1 [file Datasheet1.pdf]

Evaluation of Disinfection Protocols for Water Management in Dental Unit Waterlines

Supplement A:

Comparison Dental units: Baseline microbiological contamination

Dental Unit A1

At baseline (06/2021) in Fig.S 1, *L. pneumophila* demonstrated contamination at ≥100 CFU/100 mL in A1. *P. aeruginosa* was detected at 16 CFU/100 mL. Heterotrophic plate counts (HPC) were 18 CFU/mL at 37 °C and 0 CFU/mL at 22 °C.

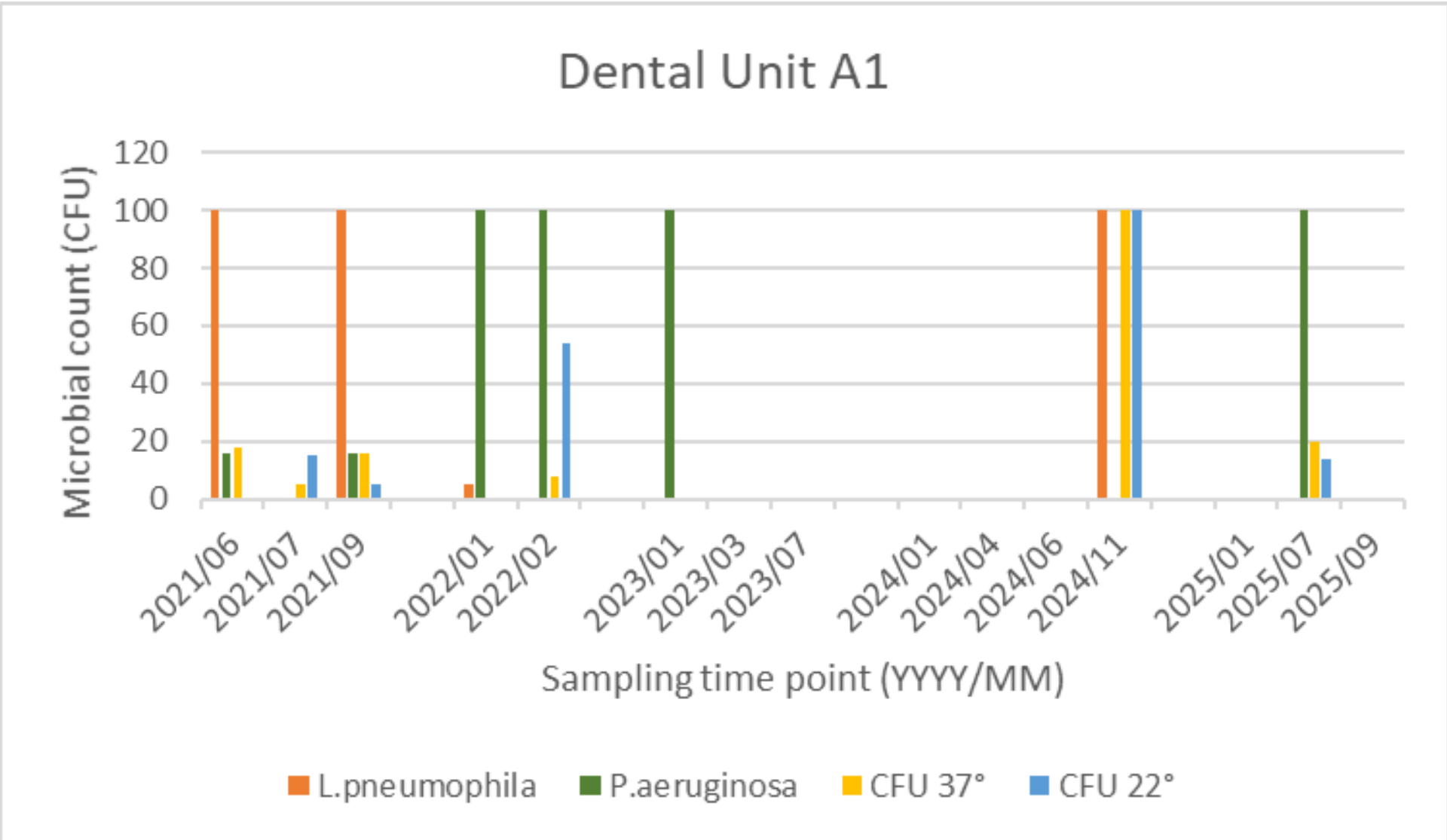

*Figure S 1: **Dental Unit 1:** showed high bacterial counts, as well for L.pneumophila as for P. aeruginosa. After the change to protocol A, contamination get less, but still occasional pops up in 2024/11 and 2025/07.*

In 07/2021, both *L. pneumophila* and *P. aeruginosa* were undetectable (0 CFU/100 mL). HPC decreased to 5 CFU/mL (37 °C) and increased slightly to 15 CFU/mL (22 °C), remaining within acceptable limits. By 09/2021, *L. pneumophila* again reached ≥100 CFU/100 mL, while *P. aeruginosa* was present at 6 CFU/100 mL. HPC values were 16 CFU/mL (37 °C) and 5 CFU/mL (22 °C). In 01/2022, *L. pneumophila* decreased to 5 CFU/100 mL; however, *P. aeruginosa* showed a pronounced increase to ≥100 CFU/100 mL. HPC values were 0 CFU/mL at both temperatures. In 02/2022, *L. pneumophila* was no longer detectable (0 CFU/100 mL), whereas *P. aeruginosa* remained at ≥100 CFU/100 mL. HPC increased to 8 CFU/mL (37 °C) and 54 CFU/mL (22 °C).

Following intervention, *L. pneumophila* remained consistently undetectable throughout the entire observation period (2023–2025). *P. aeruginosa* showed a single peak in 01/2023 (≥100 CFU/100 mL), followed by complete absence until a minor recurrence in 07/2025 (14 CFU/100 mL). HPC values were predominantly 0 CFU/mL from 2023 to mid-2024. A transient increase was observed in 11/2024, with ≥100 CFU/mL at both 37 °C and 22 °C. In 07/2025, HPC values were 20 CFU/mL (37 °C) and 14 CFU/mL (22 °C), followed by complete normalization (0 CFU/mL) in 09/2025. Overall, A1 exhibited the highest variability and was the only unit with repeated post-intervention deviations.

Dental Unit A2

At baseline (06/2021) in Fig.S 2, *L. pneumophila* was detected at ≥100 CFU/100 mL, while *P. aeruginosa* was absent (0 CFU/100 mL). HPC values were 20 CFU/mL (37 °C) and 0 CFU/mL (22 °C).

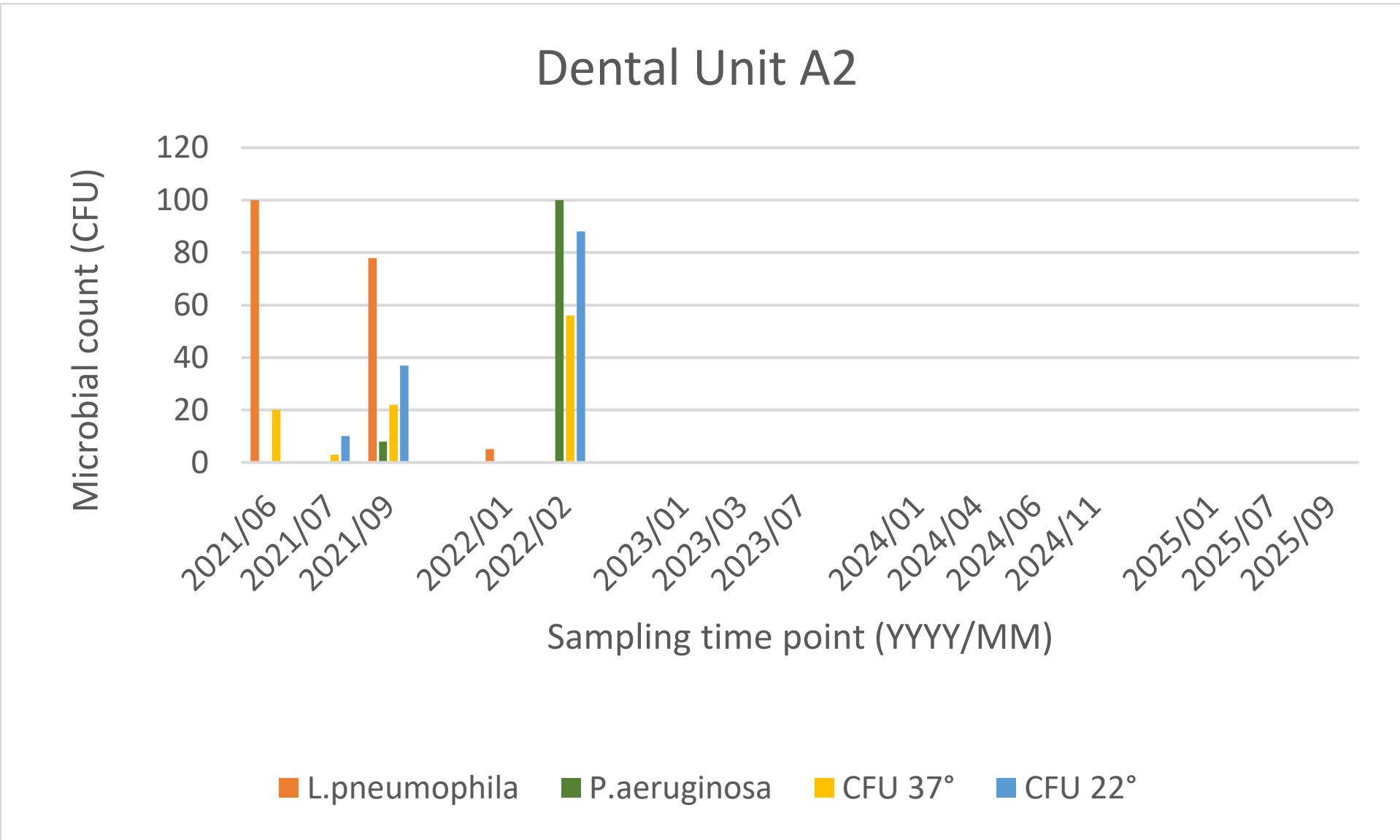

*Figure S 2: **Dental Unit 2**: High bacterial counts for L. pneumophila 2021/06 and 2022/02 for P. aeruginosa and heterotroph colony forming units 22° and colony forming units 37°, after changing protocol to standard A, no further contamination was observed*

In 07/2021, no pathogens were detected. HPC values were low (3 CFU/mL at 37 °C and 10 CFU/mL at 22 °C). In 09/2021, *L. pneumophila* was reduced to 78 CFU/100 mL, while *P. aeruginosa* was detected at 8 CFU/100 mL. HPC values increased to 22 CFU/mL (37 °C) and 37 CFU/mL (22 °C). In 01/2022, *L. pneumophila* was further reduced to 5 CFU/100 mL and

P. aeruginosa remained undetectable. HPC values were 0 CFU/mL. In 02/2022, *P. aeruginosa* reached ≥100 CFU/100 mL, while *L. pneumophila* remained undetectable. HPC values increased to 56 CFU/mL (37 °C) and 88 CFU/mL (22 °C).

After 2022, all parameters in A2 remained consistently at 0 CFU across the entire observation period (2023–2025), with no further deviations.

Dental Unit A3

At baseline (06/2021), *L. pneumophila* was detected at ≥100 CFU/100 mL, while *P. aeruginosa* was absent (Fig. S 3). HPC values were 20 CFU/mL (37 °C) and 0 CFU/mL (22 °C).

In 09/2021, both *L. pneumophila* and *P. aeruginosa* reached ≥100 CFU/100 mL. HPC values were 10 CFU/mL (37 °C) and 9 CFU/mL (22 °C). In 02/2022, *P. aeruginosa* remained at ≥100 CFU/100 mL, while *L. pneumophila* was no longer detectable. HPC values were 8 CFU/mL (37 °C) and 16 CFU/mL (22 °C).

Following intervention, *L. pneumophila* and *P. aeruginosa* were consistently undetectable throughout the entire period (2023–2025). HPC values remained at 0 CFU/mL until 11/2024, when an isolated increase was observed (88 CFU/mL at 37 °C and 98 CFU/mL at 22 °C). No further elevations were detected thereafter.

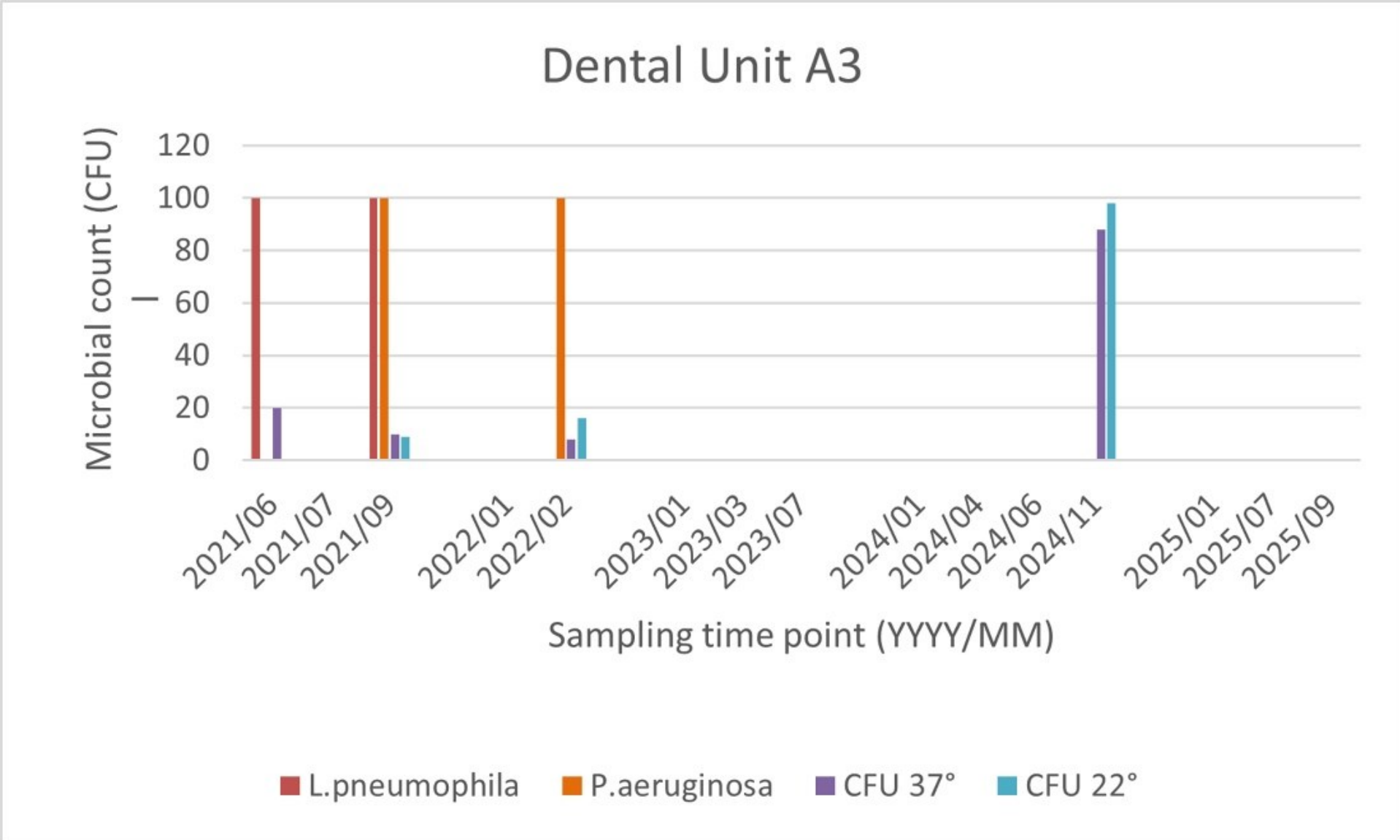

*Figure S 3: **Dental Unit A3:** High bacterial counts for L. pneumophila 2021/06 and 2021/09 and 2022/02 for P. aeruginosa, after changing protocol to standard A, minor contamination in 2024/11 was observed by heterotroph CFU 22° and CFU 37°.*

Dental Unit A4

At baseline (06/2021) in (Fig. S 4), *L. pneumophila* was detected at ≥100 CFU/100 mL, while *P. aeruginosa* was absent. HPC values were 25 CFU/mL (37 °C) and 0 CFU/mL (22 °C). In 07/2021, no pathogens were detected, and HPC values were minimal (2 CFU/mL at both temperatures). By 09/2021, *L. pneumophila* again reached ≥100 CFU/100 mL, while *P. aeruginosa* was detected at 22 CFU/100 mL. HPC values increased to 35 CFU/mL (37 °C) and 20 CFU/mL (22 °C). In 01/2022, both pathogens were absent (0 CFU/100 mL), and HPC values were 0 CFU/mL. In 02/2022, *P. aeruginosa* increased markedly to ≥100 CFU/100 mL, while *L. pneumophila* remained undetectable. HPC values were 32 CFU/mL (37 °C) and 22 CFU/mL (22 °C).

After intervention, *L. pneumophila* and *P. aeruginosa* remained consistently undetectable. A single transient HPC elevation was observed in 03/2023, reaching 80 CFU/mL (37 °C) and 96 CFU/mL (22 °C), remaining within acceptable limits. No further abnormalities were detected.

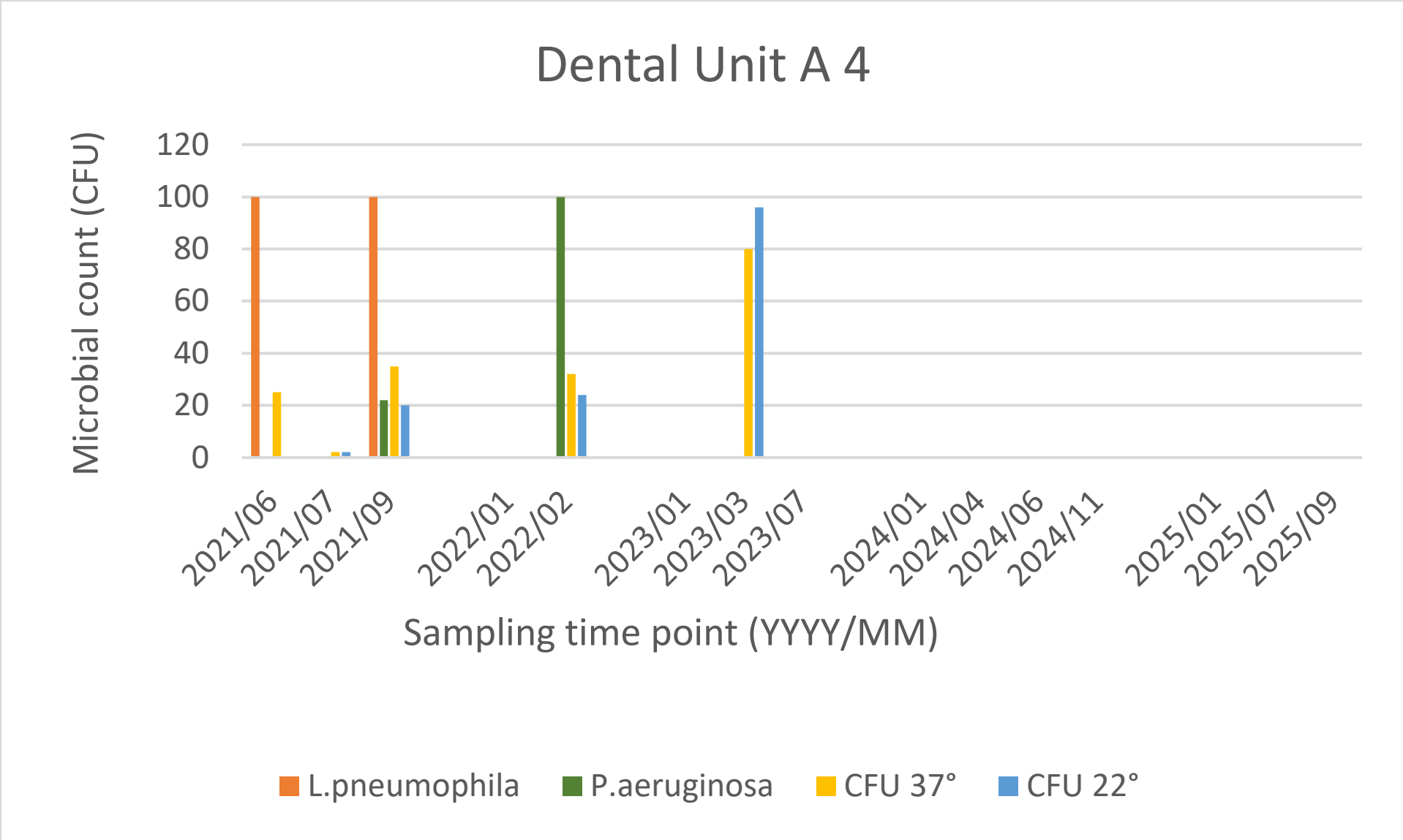

Figure S 4: Dental ***Unit A4****: High bacterial counts for L. pneumophila 2021/06 and 2021/09 and 2022/02 for P. aeruginosa, after changing protocol to standard A, minor contamination in 2023/03 was observed by heterotroph HPC°.*

Dental Unit A5

At baseline (06/2021) (Fig.S 5), *L. pneumophila* was detected at ≥100 CFU/100 mL, while *P. aeruginosa* was absent. HPC values were 31 CFU/mL (37 °C) and 0 CFU/mL (22 °C). In 09/2021, *L. pneumophila* remained at ≥100 CFU/100 mL, while *P. aeruginosa* was not detected. HPC values were low (0 CFU/mL at both temperatures). In 02/2022, *P. aeruginosa* reached ≥100 CFU/100 mL, while *L. pneumophila* was no longer detectable. HPC values increased to 36 CFU/mL (37 °C) and 68 CFU/mL (22 °C). Following intervention, both pathogens were consistently absent. In 03/2023, a minimal detection of *P. aeruginosa* (1 CFU/100 mL) was observed, accompanied by HPC values of 0 CFU/mL (37 °C) and 1 CFU/mL (22 °C). No further deviations occurred.

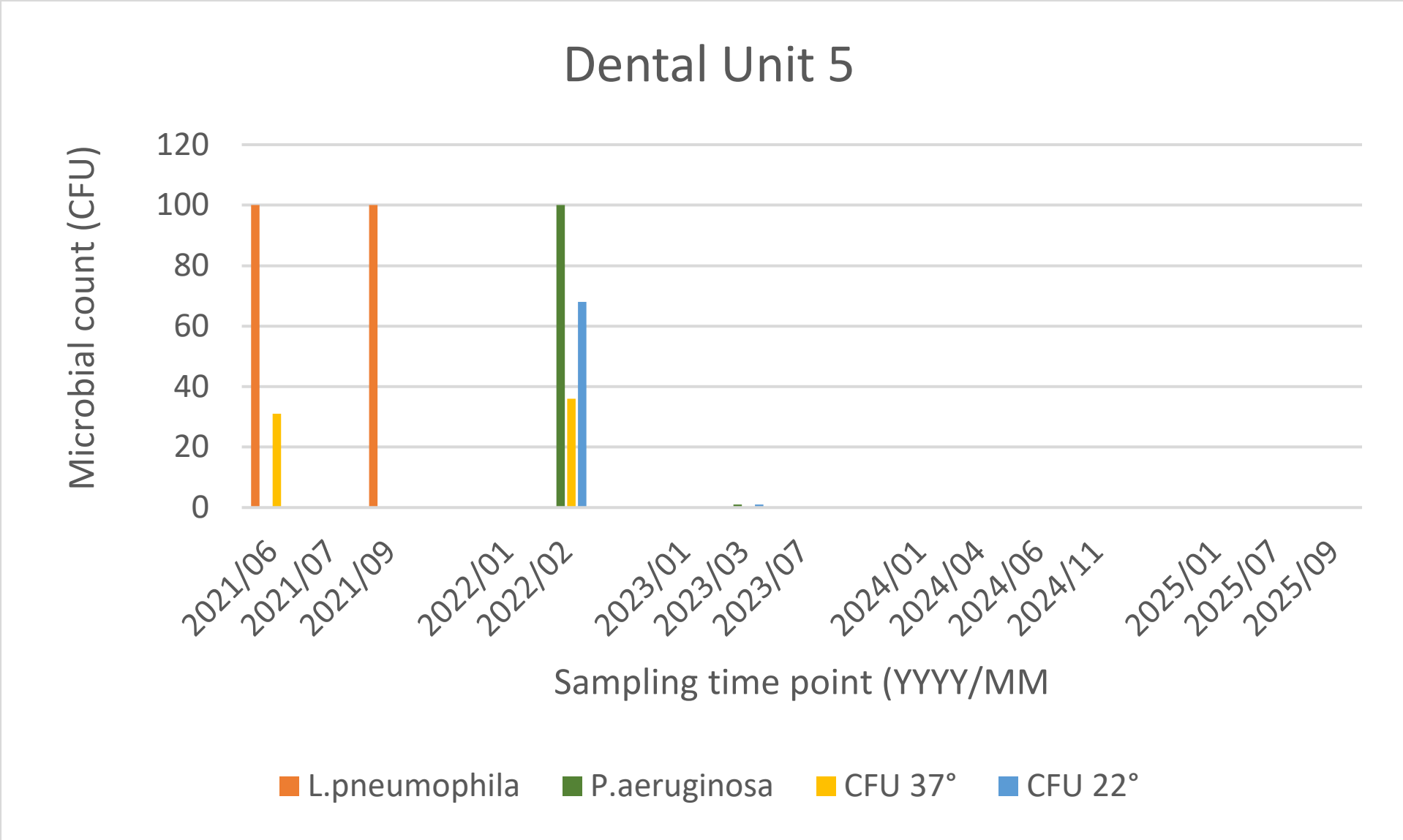

Figure S 5: ***Dental Unit 5****: High bacterial counts for L. pneumophila 2021/06 and 2021/09 and 2022/02 for P. aeruginosa, together with minor contamination by heterotroph CFU 22° and CFU37°, after changing protocol to standard A, no further contamination was observed.*

Dental Unit A6

At baseline (06/2021) (Fig. S 6) *L. pneumophila* was detected at ≥100 CFU/100 mL. HPC values were 8 CFU/mL (37 °C) and 0 CFU/mL (22 °C).

In 09/2021, *L. pneumophila* decreased to 66 CFU/100 mL, while *P. aeruginosa* increased to ≥100 CFU/100 mL. HPC values were 10 CFU/mL (37 °C) and 48 CFU/mL (22 °C). In 01/2022, *L. pneumophila* was detected at 15 CFU/100 mL, while *P. aeruginosa* remained ≥100 CFU/100 mL. In 02/2022, *P. aeruginosa* remained at ≥100 CFU/100 mL, while *L. pneumophila* was no longer detectable. HPC values were 15 CFU/mL (37 °C) and 40 CFU/mL (22 °C).

After intervention, both pathogens were consistently absent. However, a pronounced HPC increase occurred in 03/2023, reaching ≥100 CFU/mL at both temperatures, indicating transient biofilm activity. No further elevations were observed thereafter.

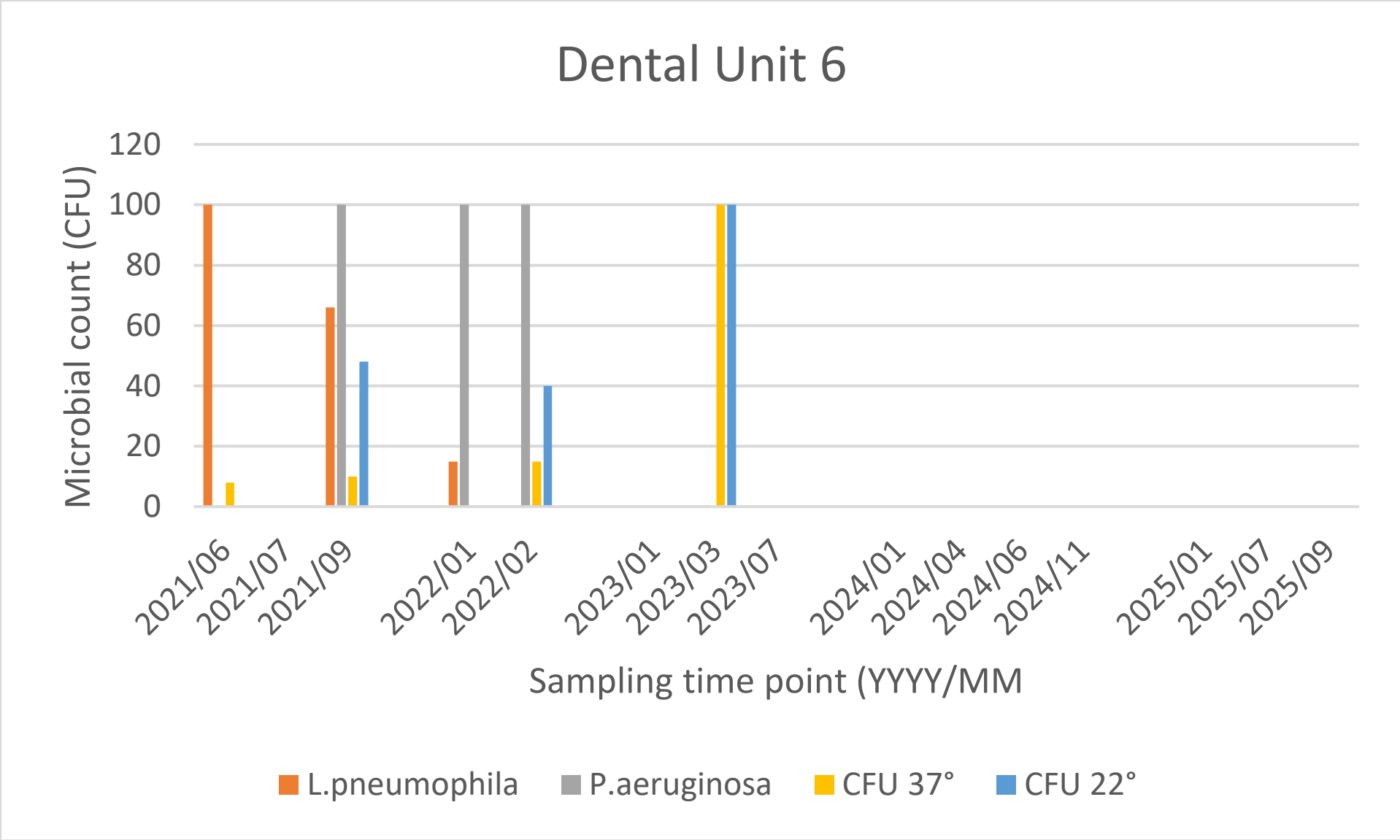

*Figure S 6: **Dental Unit 6:** High bacterial counts for L. pneumophila 2021/06 and 2021/09, 2022/01, 2022/02 for P. aeruginosa, after changing protocol to standard A, 2023/03 contamination for CFU 22° and CFU 37°was observed*

Dental Unit 7

At baseline (06/2021) (Fig. S 7), *L. pneumophila* was detected at ≥100 CFU/100 mL. HPC values were 0 CFU/mL.

In 09/2021, *L. pneumophila* and *P. aeruginosa* both reached ≥100 CFU/100 mL. HPC values were 10 CFU/mL (37 °C) and 33 CFU/mL (22 °C). In 02/2022, *P. aeruginosa* remained ≥100 CFU/100 mL, while *L. pneumophila* was undetectable. HPC values were 22 CFU/mL (37 °C) and 23 CFU/mL (22 °C).

Following intervention, both pathogens remained absent. In 03/2023, a slight increase in HPC was observed (1 CFU/mL at 37 °C and 10 CFU/mL at 22 °C). A further localized increase occurred in 11/2024, with HPC values of 63 CFU/mL (37 °C) and 33 CFU/mL (22 °C), remaining within acceptable limits.

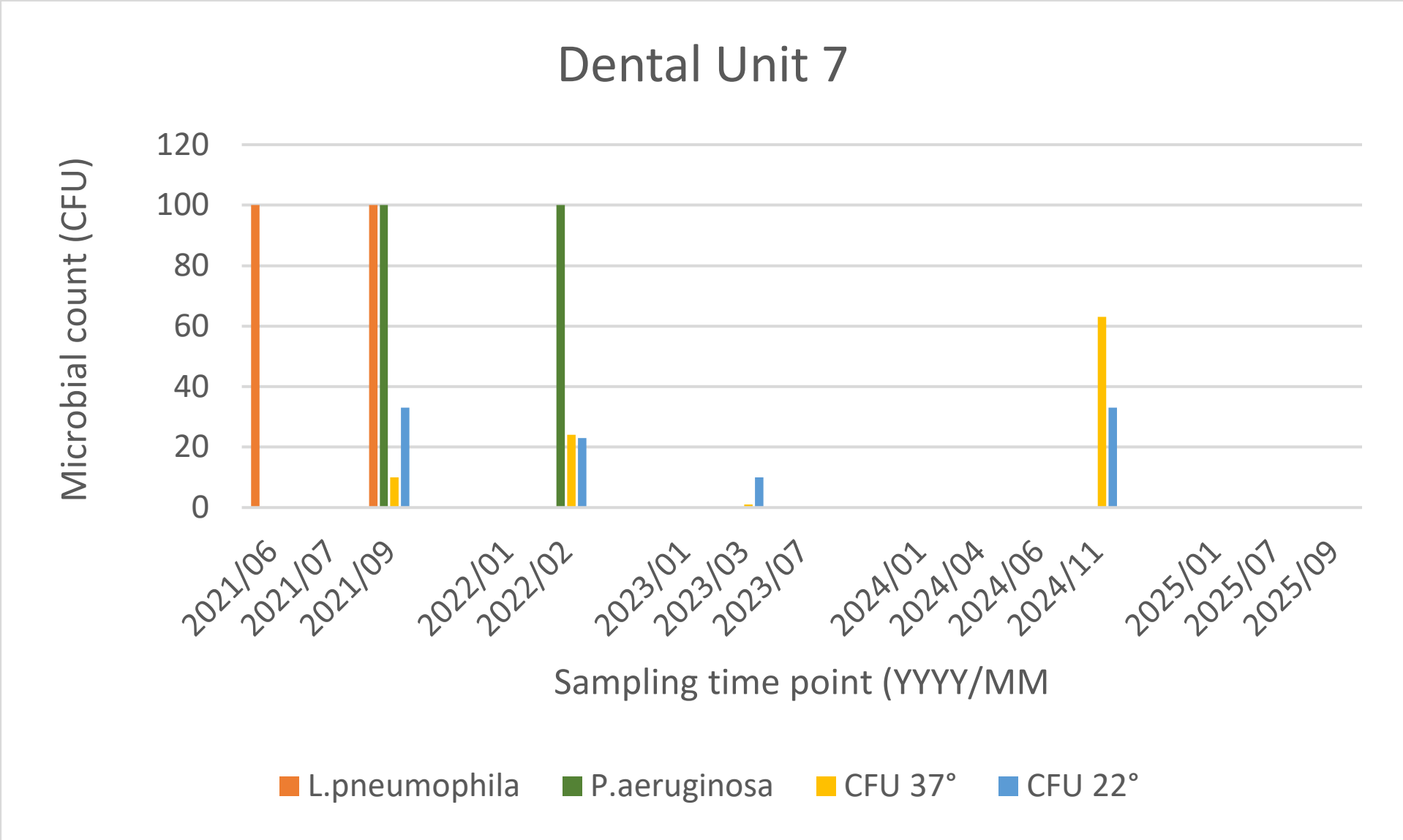

*Figure S 7: **Dental Unit 7:** High bacterial counts for L. pneumophila 2021/06 and 2021/09 and for P. aeruginosa, after changing protocol to standard A, minor contamination by HPC in 2024/11 was observed.*

Dental Unit A8

At baseline (06/2021) (Fig. S 8) *L. pneumophila* was detected at ≥100 CFU/100 mL.

In 09/2021, *L. pneumophila* was detected at 84 CFU/100 mL, while *P. aeruginosa* was absent. HPC values were 12 CFU/mL (37 °C) and 21 CFU/mL (22 °C). In 02/2022, *P. aeruginosa* reached ≥100 CFU/100 mL. HPC values were 4 CFU/mL (37 °C) and 8 CFU/mL (22 °C).

After intervention, both pathogens were absent throughout. A transient HPC increase was observed in 11/2024, reaching 77 CFU/mL (37 °C) and 42 CFU/mL (22 °C), followed by normalization.

In 07/2025, a minor increase was observed (15 CFU/mL at 37 °C and 28 CFU/mL at 22 °C).

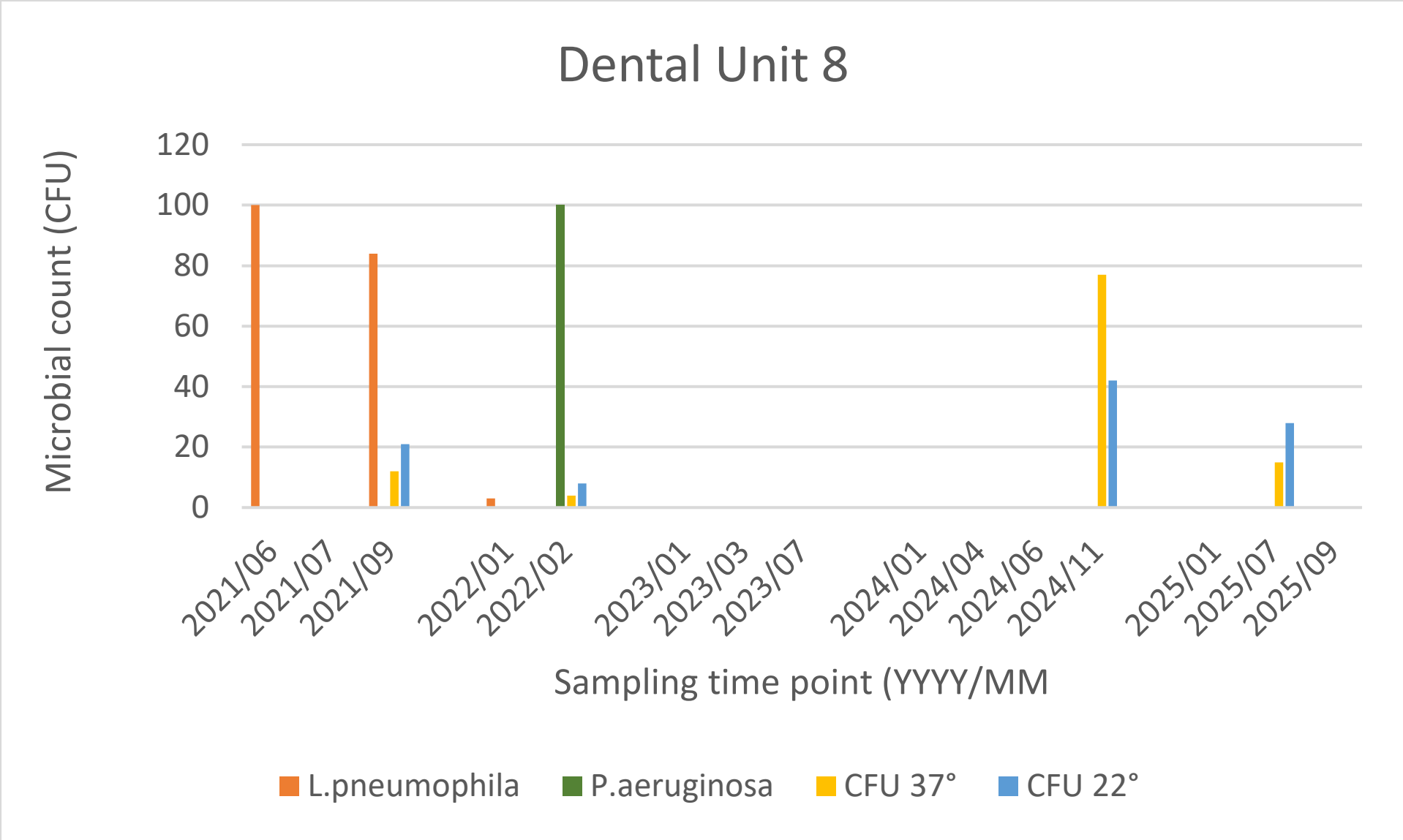

*Figure S 8:**Dental Unit 8**: High bacterial counts for L. pneumophila 2021/06 and 2021/09and 2022/02 for P. aeruginosa, after changing protocol to standard A, minor contamination by HPC was observed in 2024/11 und 2025/07.*

Dental Unit A9

At baseline (06/2021), (Fig S 9), *L. pneumophila* was detected at ≥100 CFU/100 mL. HPC values were 0 CFU/mL.

In 09/2021, *L. pneumophila* was detected at low levels (6 CFU/100 mL), while *P. aeruginosa* reached 44 CFU/100 mL. HPC values were 33 CFU/mL (37 °C) and 3 CFU/mL (22 °C). In 01/2022, *L. pneumophila* was detected at 10 CFU/100 mL, and *P. aeruginosa* reached ≥100 CFU/100 mL. In 02/2022, *P. aeruginosa* remained ≥100 CFU/100 mL, while *L. pneumophila* was absent. HPC values were 41 CFU/mL (37 °C) and 96 CFU/mL (22 °C).

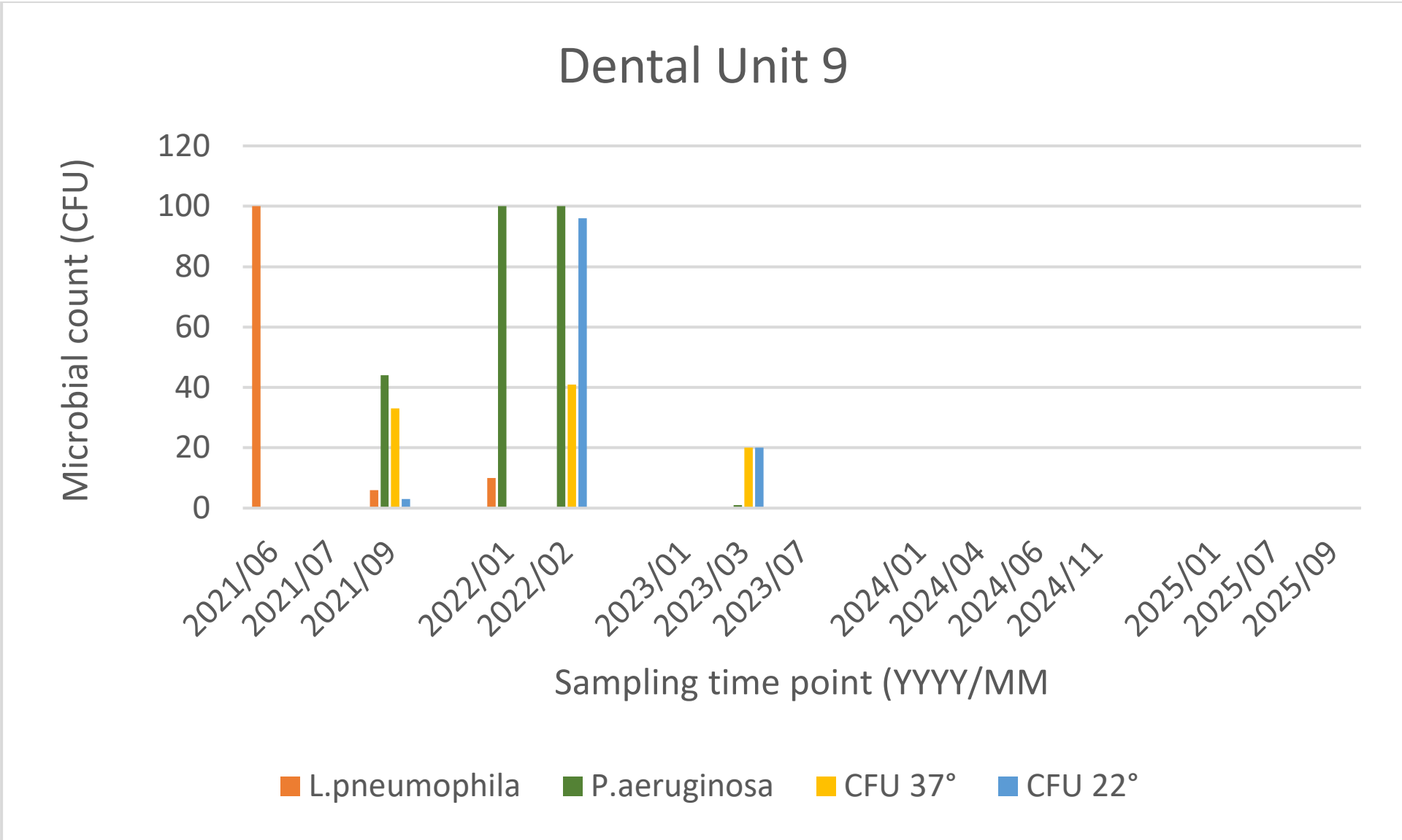

Figure S 9: ***Dental Unit 9****: High bacterial counts in for L. pneumophila 2021/06 and 2022/02 for P. aeruginosa and CFU 22°, after changing protocol to standard A, no further contamination was observed.*

Following intervention, both pathogens remained absent. In 03/2023, HPC values increased to 20 CFU/mL (37 °C and 22 °C), followed by complete normalization thereafter.

Supplement B: Evaluation of Disinfective Protocoll

Before protocol standardization, repeated contamination with high counts (over 100 CFU/ml) *L. pneumophila*. and *P. aeruginosa* was detected. Following the evaluation of different disinfection protocols and the implementation of a standardized HOCl-based automated flushing system, a marked reduction in contamination events was observed.

Supplement B:

The results for difference between effectivity of 3 disinfections solutions

No *L. pneumophila* growth was confirmed within any of the water samples in this research.

Results for P. aeruginosa and HPC count on yeast agar medium at 22°C indicate significant

differences between the disinfections, while on yeast agar medium at 37°C the differences are not statistically significant (S Tab 1).

S Table 1. Post hoc analysis and pairwise comparison of three disinfection Solutions A, B, and C

**Type of medium**	**Comparison of disinfections**	**significance**
PA	A, B	0.995 not Significant
	A, C	< 0.001 Significant
	B, C	< 0.001 Significant
Agar medium at 22 °C	A, B	0.419 not significant
	A, C	0.003 Significant
	B, C	< 0.001 Significant
Agar medium at 37 °C	A, B	0.098 not Significant
	A, C	0.993 not Significant
	B, C	0.124 not Significant
L. pneumophila	-	-

- **Legionella pneumophila:**

Not detected in a 100ml water sample after any form of disinfection (during Evaluation) (Fig. S10).

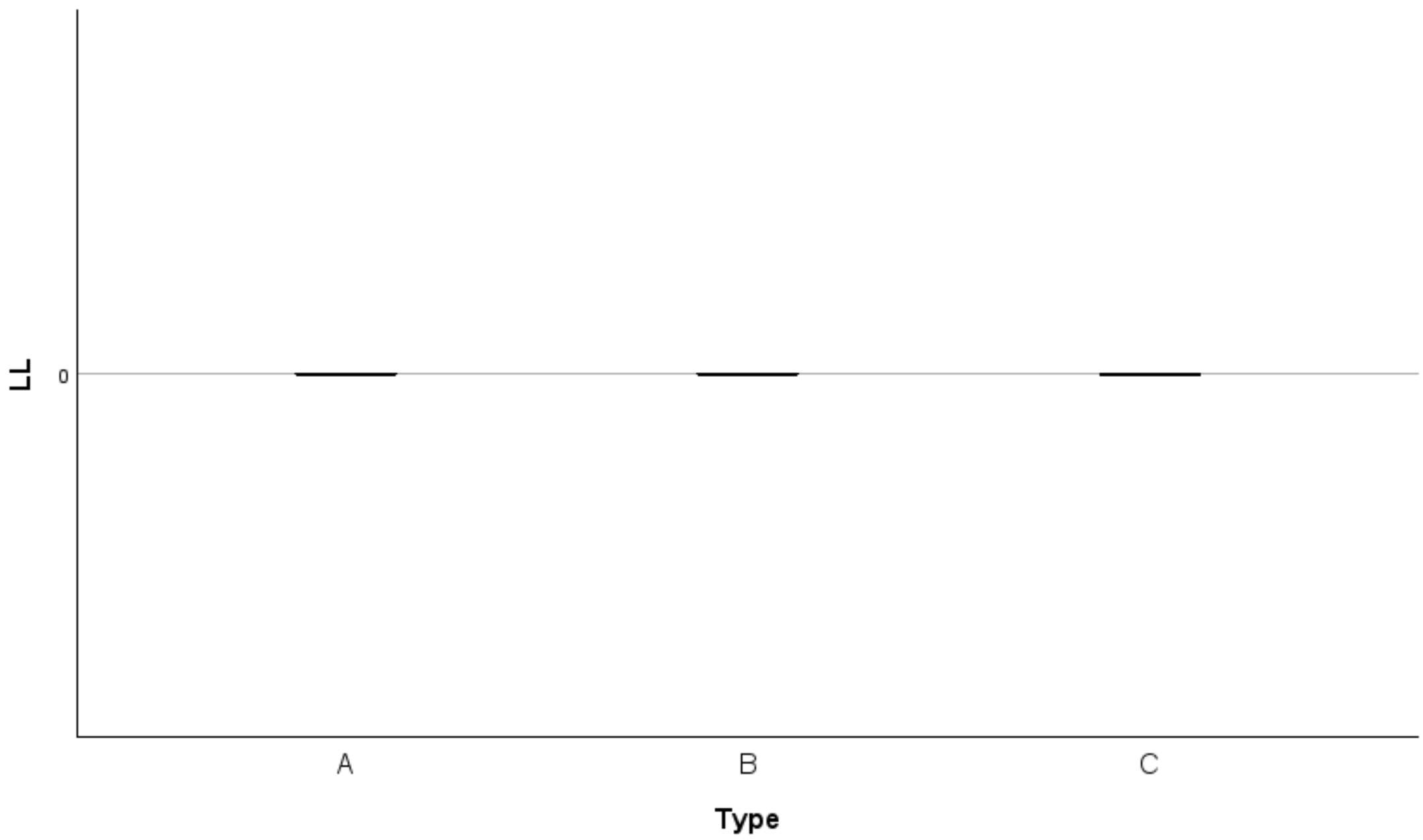

Figure S 10: Legionella pneumophila: no detection during the evaluation phase.

- **Pseudomonas aeruginosa**

P-value is < 0.001, indicating that there are significant differences between Protocol A and B, with sufficient reduction of *P. aeruginosa*, and protocol C in disinfections (Fig.S 11).

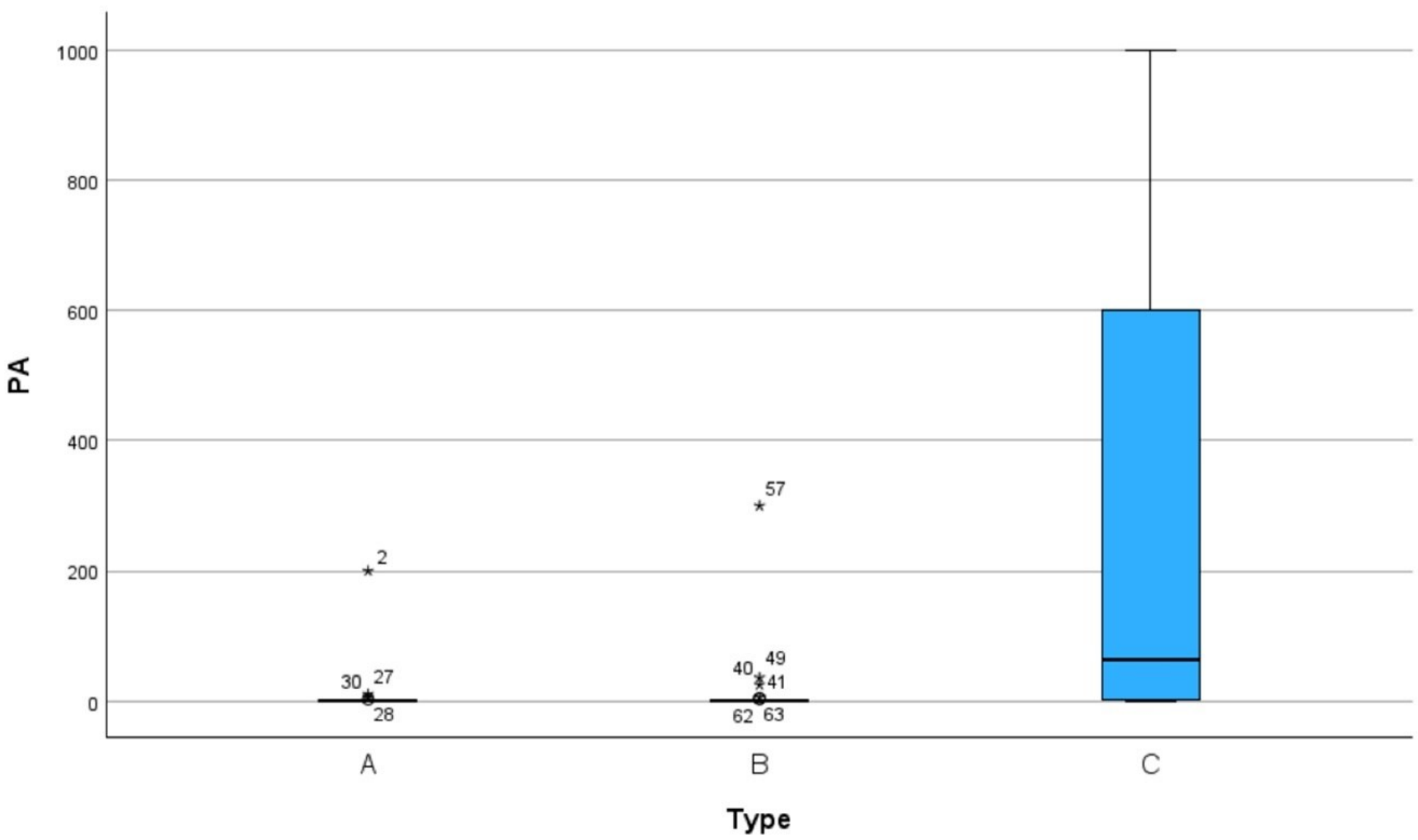

Figure S 11: ***Evaluation for P. aeruginosa****: Protocol A show highest bacterial reduction, Protocol B similar and Protocol C show high bacterial contamination with over 100 CFU.*

- **Heterotrophic Plate count (HPC) on yeast agar medium at 22°C**

P-value is < 0.001, suggesting that there are significant differences between Protocol A and B, with sufficient reduction of HPC, and protocol C in disinfections (Fig.S 12).

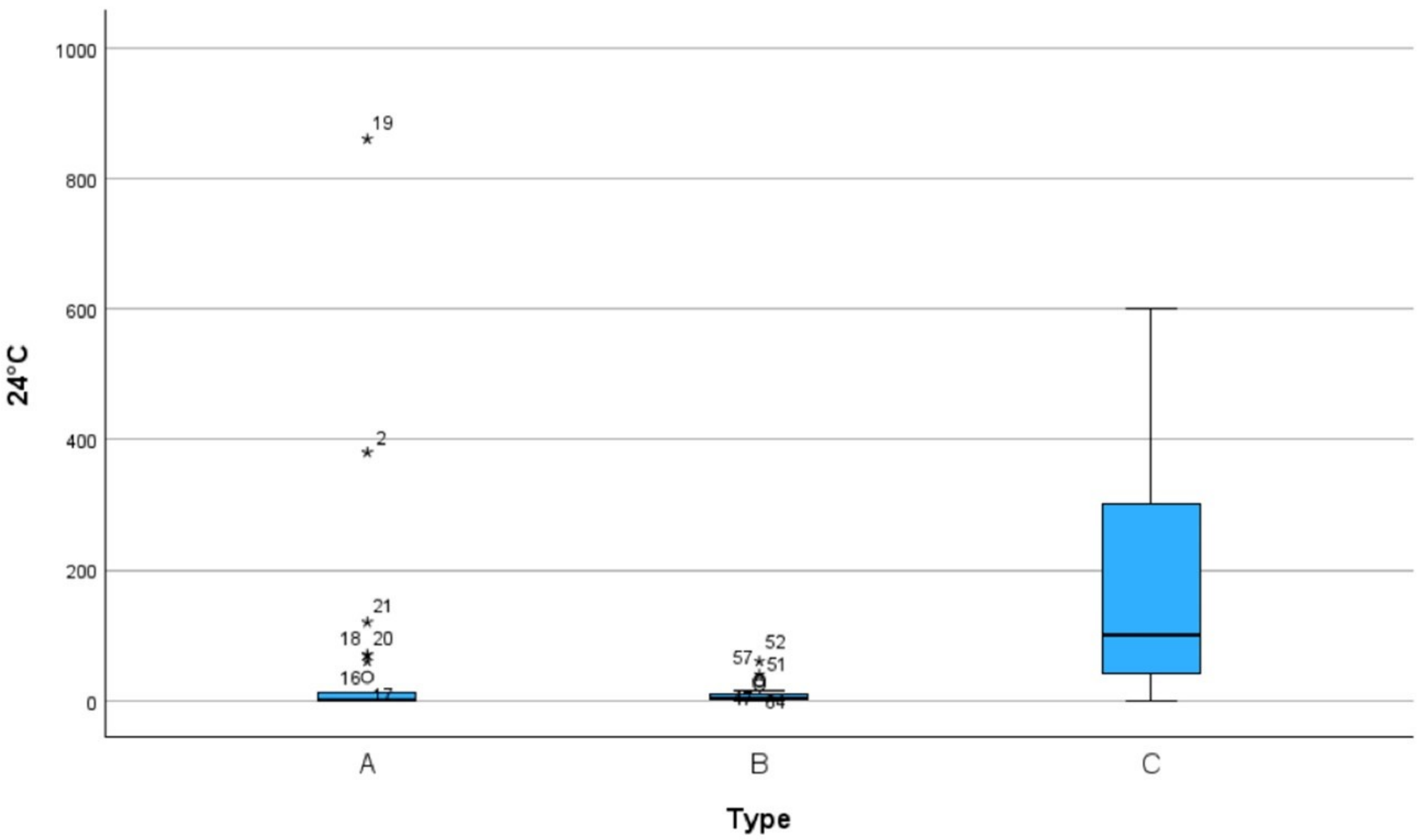

*Figure S 12: **Evaluation heterotrophic bacterial count**: Protocol A show highest bacterial reduction, Protocol B similar and Protocol C show high bacterial contamination with over 100 CFU.*

- **HPC on yeast agar medium at 37°C**

P-value of 0.069, indicating that the result is not statistically significant.

Regarding retrograde contamination, there are no significant differences between morning and afternoon samples concerning CFU count at any medium. All Tables and Figures available in Supplements (Fig. S13).

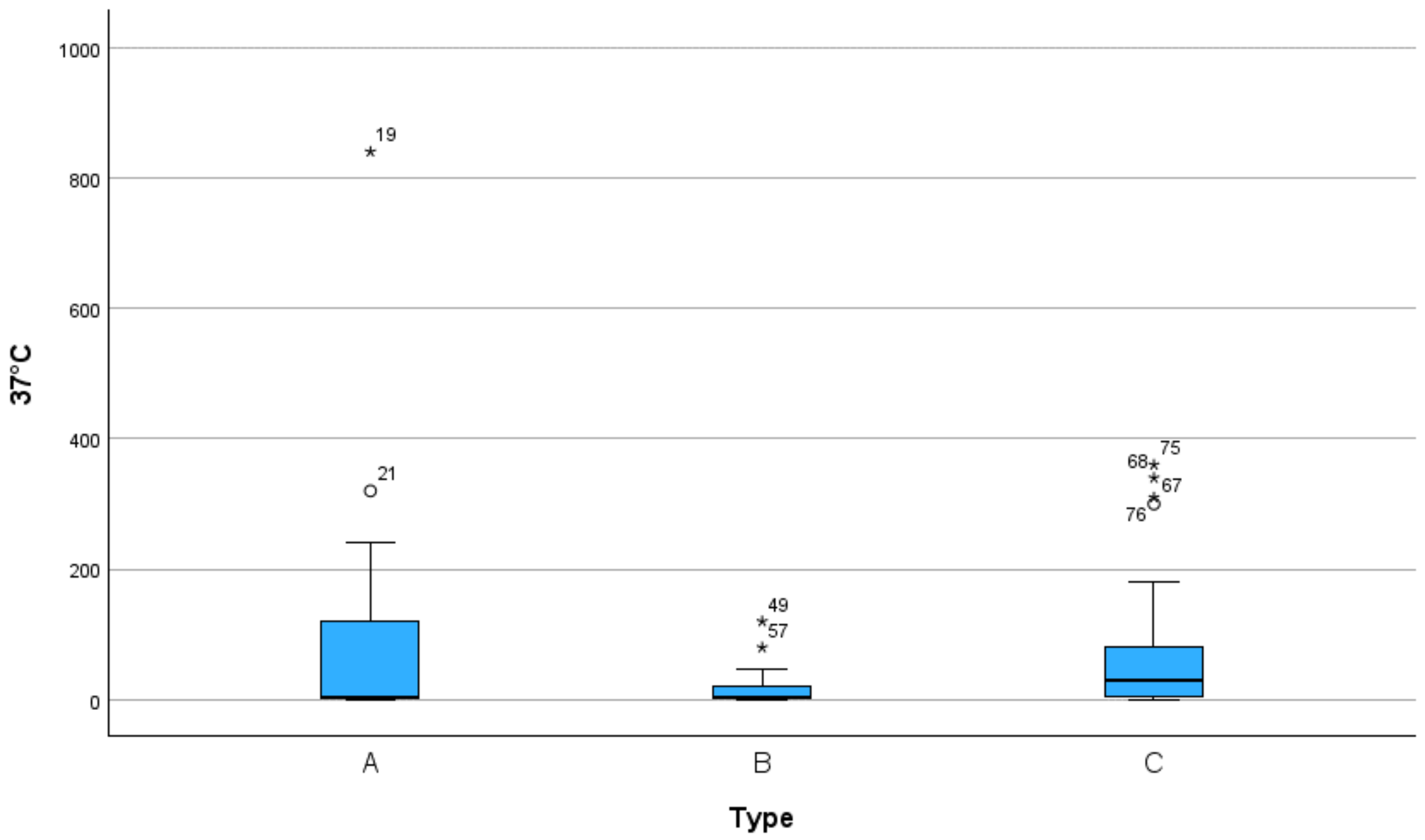

*Figure S13: **Evaluation heterotrophic bacterial count**: Protocol A and C show highest bacterial count with over 100 CFU. Protocol B minor contamination.*

Table S 2. Post hoc analysis and pairwise comparison regarding difference at CFU for each disinfection at morning and afternoon.

**Type of medium**	**Difference at CFU for each disinfection at morning and afternoon**	**significance**
PA	A morning- A afternoon	1 not significant
	B morning- B afternoon	1 not significant
	C morning- C afternoon	0.907 not significant
Agar medium at 22 °C	A morning- A afternoon	0.607 not significant
	B morning- B afternoon	1 not significant
	C morning- C afternoon	0.994 not significant
Agar medium at 37 °C	A morning- A afternoon	0.726 not significant
	B morning- B afternoon	1 not significant
	C morning- C afternoon	1 not significant
L. pneumophila	-	-

With exception in heterotrophic bacterial count at 37° storage, during the evaluation phase, the increased hypochlorite concentration used in Protocol B did not result in a meaningful additional reduction in bacterial contamination compared with the standard Protocol A. But it was associated with technical impairments affecting specific components of dental units. Protocol C (HOCl combined with H_2O_2) exhibited the lowest overall effectiveness in microbiological control.
